# Hairless-binding deficient *Suppressor of Hairless* alleles reveal Su(H) protein levels are dependent on complex formation with Hairless

**DOI:** 10.1371/journal.pgen.1006774

**Published:** 2017-05-05

**Authors:** Heiko Praxenthaler, Anja C. Nagel, Adriana Schulz, Mirjam Zimmermann, Markus Meier, Hannes Schmid, Anette Preiss, Dieter Maier

**Affiliations:** Institute of Genetics (240), University of Hohenheim, Stuttgart, Germany; Cincinnati Children's Hospital Medical Center, UNITED STATES

## Abstract

Cell fate choices during metazoan development are driven by the highly conserved Notch signalling pathway. Notch receptor activation results in release of the Notch intracellular domain (NICD) that acts as transcriptional co-activator of the DNA-binding protein CSL. In the absence of signal, a repressor complex consisting of CSL bound to co-repressors silences Notch target genes. The *Drosophila* repressor complex contains the fly CSL orthologue Suppressor of Hairless [Su(H)] and Hairless (H). The Su(H)-H crystal structure revealed a large conformational change within Su(H) upon H binding, precluding interactions with NICD. Based on the structure, several sites in Su(H) and H were determined to specifically engage in complex formation. In particular, three mutations in Su(H) were identified that affect interactions with the repressor H but not the activator NICD. To analyse the effects these mutants have on normal fly development, we introduced these mutations into the native *Su(H)* locus by genome engineering. We show that the three H-binding deficient *Su(H)* alleles behave similarly. As these mutants lack the ability to form the repressor complex, Notch signalling activity is strongly increased in homozygotes, comparable to a complete loss of *H* activity. Unexpectedly, we find that the abundance of the three mutant Su(H) protein variants is altered, as is that of wild type Su(H) protein in the absence of H protein. In the presence of NICD, however, Su(H) mutant protein persists. Apparently, Su(H) protein levels depend on the interactions with H as well as with NICD. Based on these results, we propose that *in vivo* levels of Su(H) protein are stabilised by interactions with transcription-regulator complexes.

## Author summary

Notch signalling activity plays a major role in determining cell fates. Notch signals are transduced into gene expression changes by the transcription factor CSL and the activated Notch receptor intracellular domain (NICD). CSL can also function as a transcriptional repressor, depending on its bound cofactors. In *Drosophila*, repression of Notch target genes involves the CSL homologue Suppressor of Hairless [Su(H)] and the Notch antagonist Hairless (H). H binding to Su(H) excludes simultaneous NICD binding. Based on structural information, amino acids important for Su(H)-H interactions were mutated, generating Su(H) molecules that still bind NICD but no longer H, thereby preventing repressor but not activator complex formation. Three such mutations were introduced into the native *Su(H)* locus to analyse their consequences on fly development *in vivo*. All three alleles are homozygous lethal, demonstrating the essential role of Su(H) as repressor during fly development. Moreover, all three H-binding deficient *Su(H)* alleles show marked Notch gain of function. Unexpectedly, protein abundance of mutant Su(H) variants is reduced due to the loss of interactions with H. Moreover, we find that Su(H)-NICD interaction increased mutant Su(H) protein levels. Taken together, we propose that Su(H) protein is stabilised *in vivo* by interactions with transcription-regulator complexes.

## Introduction

Cell-to-cell communication is essential to development in higher animals and relies in part on the Notch signalling cascade which specifies the lineage of cells in a multitude of tissues [1,2]. As a consequence of activating Notch signalling—and subsequently Notch target genes—cells are directed into specific cell fates. The Notch signalling pathway is highly conserved, it is found from worms to insects and mammals, and defects in the Notch pathway are involved in a number of congenital human diseases and several types of human cancers [3].

The principles of Notch signal transduction are rather simple: interactions of the Notch receptor with its ligands result in cleavage of Notch, nuclear translocation of its intracellular domain (NICD, for *Notch Intracellular Domain*), and assembly of a transcriptional activator complex with the DNA-binding protein CSL and the co-activator Mastermind (Mam) [4,5]. CSL is the initialism for the respective protein orthologues from human, *D*. *melanogaster*, and *C*. *elegans*, respectively (human C-promoter Binding Factor 1 [**C**BF1] also named RBPJ, Suppressor of Hairless [**S**u(H)] and lin-12 and Glp-1 phenotype [**L**ag1]). In the absence of a Notch signal, a repressor complex consisting of CSL and co-repressors silences transcription from Notch target genes. Most mammalian co-repressors compete with NICD for binding to a hydrophobic pocket within the beta-trefoil domain (BTD) of CSL [5–7]. In contrast, in *Drosophila* a protein named Hairless (H) binds the C-terminal domain (CTD) of Su(H) at sites that are distinct from NICD binding [8–11]. H recruits the general co-repressors Groucho and C-terminal binding protein, resulting in transcriptional silencing of Notch target genes [12–15].

The model system *Drosophila melanogaster* has been extensively used for systematic analysis of Notch signalling, and in particular, our group has used *Drosophila* to study the Notch repressor complex *in vivo*. Recently, the crystal structure of the core Notch repressor complex, containing Su(H) and H bound to DNA, was determined [11]. Strikingly, H binds deeply into the hydrophobic core of the CTD of Su(H), thereby causing a major conformational change in Su(H) that is incompatible with NICD binding. The contacts between Su(H) and H are mostly hydrophobic in nature. In order to disrupt formation of the repressor, but not the activator complex, several alanine substitutions were introduced into Su(H). A double mutation (L445A/L514A) and two triple mutations (L434A/L445A/L514A and L445A/L514A/F516A) were shown to specifically disrupt H binding while still allowing NICD interactions as well as activator complex formation. The effects these mutations had on the biological activity of Su(H) were investigated in cell culture assays and overexpression studies [11].

In this work we addressed the question, how is Notch signalling affected *in vivo* when Su(H) is incapable of binding to H, and therefore unable to act as a repressor, but is capable in activator complex formation. Hence, we set out to analyse the effects of the three H-binding deficient Su(H) mutations on normal fly development. To this end, we replaced the *Su(H)* locus with mutant versions of *Su(H)* by genome engineering. The resultant *Su(H)* alleles *Su(H)*^*LL*^, *Su(H)*^*LLF*^, and *Su(H)*^*LLL*^ were subsequently analysed in detail. As expected for *Su(H)* alleles that are unable to bind to H, the three alleles are recessive lethal, demonstrating that H interactions are imperative to Su(H) function in the fly. Despite quantifiable and statistically significant differences in protein binding strength *in vitro* [11], the three alleles behave very similarly *in vivo*. Due to the inability to form repressor complexes, Notch signalling activity is strongly increased in homozygotes, similar to a complete loss of H activity. Unexpectedly, we note a dependence of the abundance of Su(H) protein on H protein binding. Specifically, the protein levels of the H-binding deficient mutants appeared strongly reduced in larval tissue, which was similar to the results observed for the wild type isoform in the absence of H. These data suggest that Su(H) protein is stabilised by forming a complex with H. An analogous stabilisation of Su(H) was observed when Su(H) was engaged in the activator complex. Taken together, as both, H and NICD, are involved in nuclear shuttling of Su(H), the stability of Su(H) may depend on subcellular localisation, which will be addressed in future studies.

## Results

### Generation of H-binding deficient *Su(H)* alleles by genome engineering

**Fig 1:**
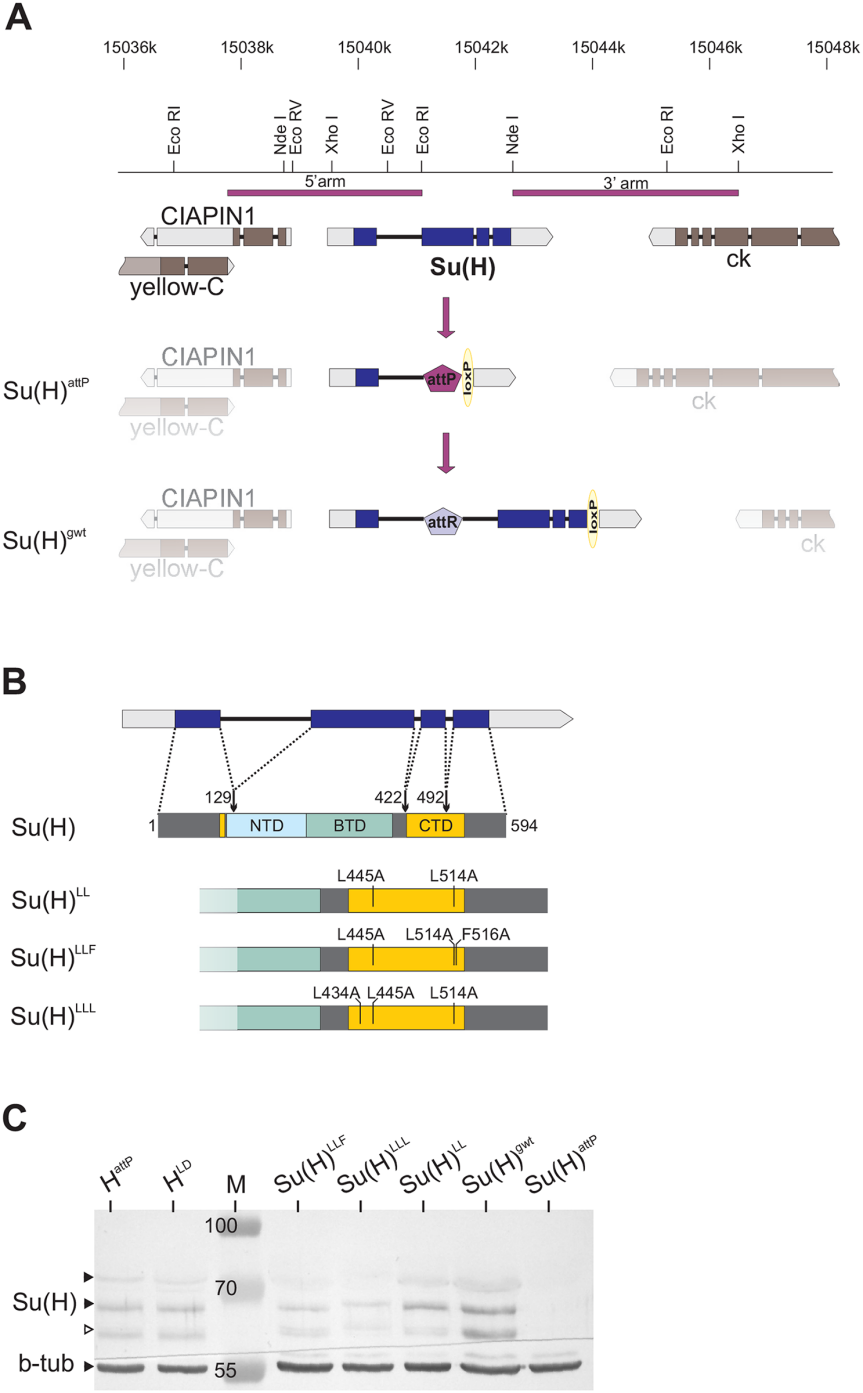
Generation of new *Su(H)* alleles deficient in H-binding by genome engineering. **(A)** The *Su(H)* locus is located on the left arm of chromosome 2; it is flanked by the genes *CIAPIN1* and *yellow-C* on the distal and *crinkled* (*ck*) on the proximal side (according to http://www.flybase.org, released 2016_02). The respective transcripts are depicted: exons are boxed, coding sequences are in dark, introns are presented as a dash; direction of transcription is indicated. Above is a restriction map with the relevant restriction sites used for cloning. Two genomic fragments, covering the 5’arm and the 3’arm, were used for homologous replacement of the locus, eventually resulting in the allele *Su(H)*^*attP*^. This served as founder line for the integration of wild type and mutant constructs as shown for *Su(H)*^*gwt*^.**(B)** The Su(H) protein consist of 594 amino acids and contains three important domains, NTD (N-terminal domain), BTD (beta-trefoil domain) and CTD (C-terminal domain). The scheme shows their relationship to the coding exons: numbers represent the amino acid position upstream of each intron. Below, the three mutants are shown that were generated within the CTD, affecting H protein binding. **(C)** Protein extracts derived from homozygous larvae of the given genotype reveal expression of Su(H) protein which, however, is reduced in the mutants. The lowest band presumably stems from degradation (open arrowhead). Beta-tubulin (b-tub) served as loading control. M, prestained protein ladder; approximate size is given in kDa.

Previously, we have shown that specific mutations in the C-terminal domain (CTD) of Su(H) significantly perturb binding interactions between Su(H) and H, thereby affecting repressor complex formation [11]. With the goal to study Su(H) proteins that are deficient in H binding *in vivo*, we generated mutant fly lines by genome engineering as outlined previously [16,17]. In the founder line *Su(H)*^*attP*^, most of the coding region was replaced by an attP landing site that served the introduction of constructs coding for the Su(H) replacement mutants *Su(H)*^*LL*^ (L445A/L514A), *Su(H)*^*LLF*^ (L445A/L514A/F516A) and *Su(H)*^*LLL*^ (L434A/L445A/L514A) (Fig 1 and S1 Fig). As a control, genomic wild type DNA constructs were introduced in *Su(H)*^*gwt*^, duplicating parts of the first intron (Fig 1A and S1 Fig), in order to not tamper with the adjacent splice acceptor of intron 1. Correct splicing was confirmed by RT-PCR (S1E Fig). Moreover, the *Su(H)*^*gwt*^ flies were indistinguishable from wild type (S2 Fig). As the mutant constructs lacked the third and/or second intron due to technical reasons, the relevant control constructs were also established and they behaved similar to the control *Su(H)*^*gwt*^ (S2 Fig). Prior to further analyses, the *white*^+^ marker gene and additional vector sequences were floxed out in all the lines (S1 Fig).

### H-binding deficient *Su(H)* alleles show characteristics of a gain of Notch activity

**Fig 2:**
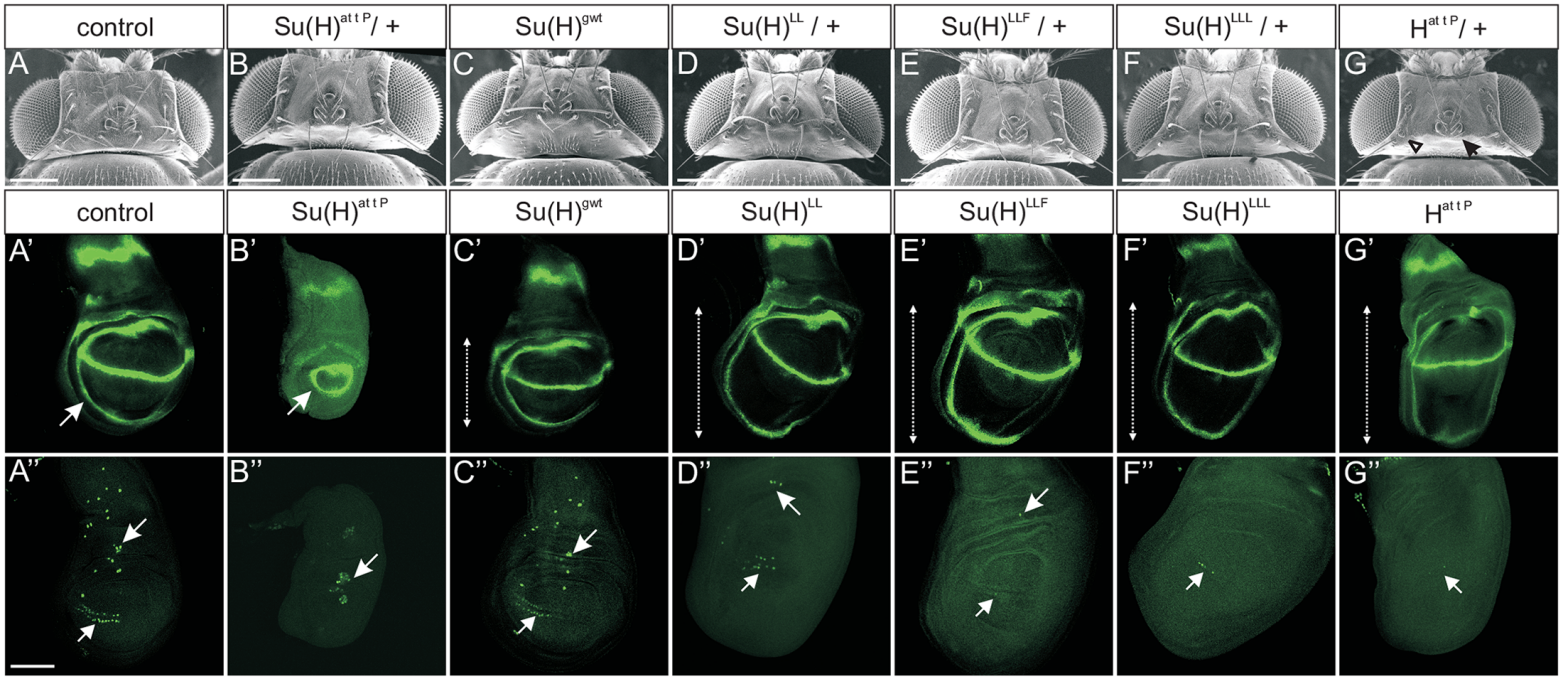
H-binding deficient *Su(H)* alleles gain Notch activity. **(A-G)** Scanning electron micrographs of fly heads with the given genotype. All heterozygous *Su(H)* mutant alleles appear like the controls. In contrast, heterozygous *H*^*attP*^ /+ flies typically lack several macrochaetae or display shaft to socket transformations (arrow and arrowhead, respectively, point to an example). Heterozygotes are in Oregon R background; Oregon R served as wild type control. Size bar represents 200μm in (A-G). **(A’-G”)** Wing imaginal discs derived from homozygous larvae of the given genotype were stained for Wingless (Wg, green, **A’-G’**) or Hindsight (Hnt, green, **A”-G”**) expression. Compared to **(A’)** *y*^*1*^ *w*^*67c23*^ control and to **(C’)** *Su(H)*^*gwt*^, the wing blade of *Su(H)*^*attP*^ homozygotes is very small **(B’)**. It is encircled by the Wg inner ring staining (arrow), and completely lacks Wg expression along the dorso-ventral boundary. **(D’-F’)** In contrast to *Su(H)*^*attP*^, wing blades in the H-binding deficient *Su(H)* mutants **(D’)** *Su(H)*^*LL*^, **(E’)** *Su(H)*^*LLF*^, or **(F’)** *Su(H)*^*LLL*^ are hypertrophied (double headed arrow), typical of a Notch gain of function. **(G’)** A similar phenotype is observed in the *H*^*attP*^ null allele. **(A”, C”)** Hnt protein is enriched in sensory organ precursors that are being selected in third instar larval wing discs to eventually form the macrochaetae on the thorax and the anterior wing margin [22] (arrow and arrowhead, respectively, point to examples). **(B”)** In the *Su(H)*^*attP*^ null mutant, sensory organ precursors are no longer singled out and appear as clusters (arrow). **(D”-G”)** Gain of Notch activity results in a reduced number of sensory organ precursors as seen in all three H-binding deficient *Su(H)* alleles **(D”-F”)**, and alike in the homozygous *H*^*attP*^ null allele **(G”)**. Size bar in (A”) represents 100μm in (A’-G”).

*Su(H)*^*attP*^ is expected to be a complete null allele and no Su(H) protein was detected in homozygous mutant larvae (Fig 1A and 1C). Accordingly, *Su(H)*^*attP*^ homozygotes died before pupation. Mutant larvae displayed small wing imaginal discs typical of a *Su(H)* loss of function [18], almost completely lacking the presumptive wing blade and the dorso-ventral boundary expression of Wingless (Wg) protein (Fig 2A’ and 2B’). As the three new mutant alleles *Su(H)*^*LL*^, *Su(H)*^*LLF*^ and *Su(H)*^*LLL*^ carry specific missense mutations, they were expected to express Su(H) protein. A conspicuous reduction was observed, however, indicative of some instability. Curiously, wild type Su(H) protein levels were similarly reduced in *H* mutant alleles (Fig 1C and S1 Fig; see below). The three alleles *Su(H)*^*LL*^, *Su(H)*^*LLF*^ and *Su(H)*^*LLL*^ were larval to pupal lethal in homo- and hemizygosis, demonstrating the pivotal role of Su(H) as a repressor for normal fly development. The heterozygous adults had the wild type appearance of the controls (Fig 2A and 2C–2F and S2 Fig). Homozygous mutant larvae developed enlarged wing imaginal discs with little variance amongst the three. Tissue hyperplasia is a typical sign for Notch hyperactivity [19,20]. Wg staining appeared normal or even enhanced along the dorso-ventral boundary (Fig 2C’–2F’). Overall, the mutant wing discs matched those of the homozygous *H*^*attP*^ null allele (Fig 2G’), indicative of a gain of Notch activity. This phenotype might be expected as a consequence of a loss of H binding, entailing the inability to repress Notch target genes. In this case, Notch signalling should generally be overactive in the *Su(H)*^*LL*^, *Su(H)*^*LLF*^ or *Su(H)*^*LLL*^ mutants, which we addressed by analysing sensory organ precursor (SOP) formation.

SOPs are singled out by Notch mediated lateral inhibition from proneural fields. In the absence of Notch activity, too many SOPs form [18,21,22]. The SOP, however, is protected by Su(H)-H mediated repression from epidermal fate [23]. This was exactly the pattern observed—SOP clusters in *Su(H)*^*attP*^ mutant discs (Fig 2B”), and no or sporadic SOPs in discs of any of the three H-binding deficient mutants compared to controls (Fig 2A”–2F”). The phenotypes again matched those of the *H*^*attP*^ mutant (Fig 2G”). We frequently observed more SOPs in *Su(H)*^*LL*^ compared with the other two mutants, suggesting some residual activity with regards to H binding and in agreement with the biochemical assays [11].

**Fig 3:**
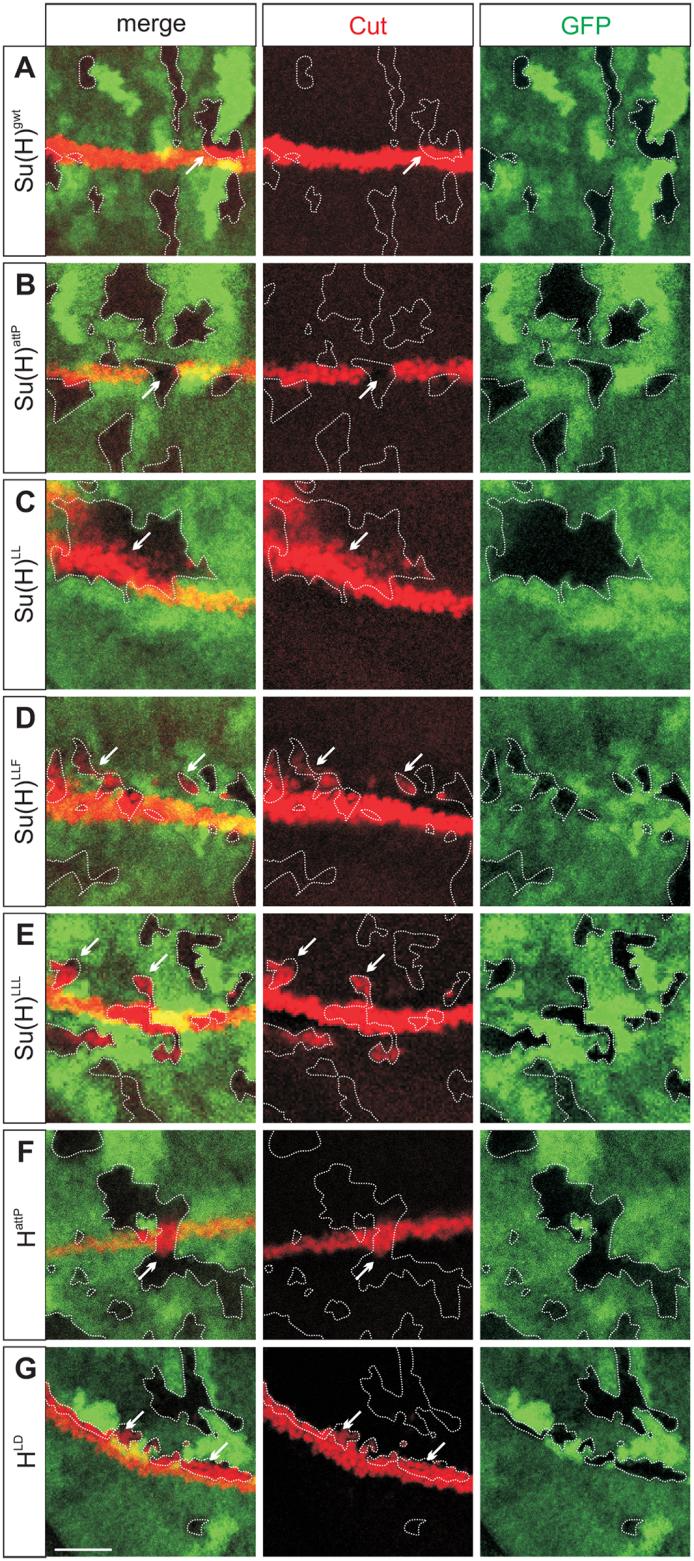
Clonal analysis of the new *Su(H)* alleles. Consequences of the *Su(H)* mutations on Notch activity were assessed by the expression of Cut (red) in cell clones homozygous for the given allele. Mutant clones are marked by the absence of GFP (green) and are surrounded by a dashed line. **(A)** Normally, Cut expression is confined to the dorso-ventral boundary; this is not affected in control clones of *Su(H)*^*gwt*^. **(B)** *Su(H)*^*att*P^ homozygous cells are devoid of Su(H) and subsequently show no Cut expression (arrow). **(C-E)** In contrast, H-binding deficient *Su(H)* mutations result in the ectopic induction of Cut expression, as seen in *Su(H)*^*LL*^ **(C)**, *Su(H)*^*LLF*^ **(D)**, and *Su(H)*^*LLL*^ cell clones **(E)**. **(F-G)** Likewise, induction of Cut expression is observed in cells homozygous for *H*^*attP*^ **(F)** or *H*^*LD*^ **(G)**, i.e. lacking the Notch antagonist H altogether and expressing a Su(H)-binding defective isoform, respectively. Accordingly, Cut is induced in homozygous cells abutting the margin. Arrows point to examples. Size bar represents 25μm in all panels.

We next induced homozygous mutant cell clones in an otherwise heterozygous background and analysed the expression of Cut (Fig 3) serving as a read out for Notch signalling activity [24–26]. Cut is normally expressed along the dorso-ventral boundary in a stripe about three cells wide (Fig 3A) [27]. While a loss of Su(H) activity resulted in the absence of Cut expression (Fig 3B), cells homozygous for any of the three H-binding deficient *Su(H)* alleles turned on Cut expression ectopically (Fig 3C–3E). Similarly, increased Cut expression was observed in *H*^*attP*^ or *H*^*LD*^ mutant cells, the latter specifically affecting H-Su(H) binding [8,17] (Fig 3F and 3G). The expression pattern of the Notch target gene *wingless (wg)* [24–26] was altered in a similar way (S3 Fig). These data support the notion that the three alleles *Su(H)*^*LL*^, *Su(H)*^*LLF*^ and *Su(H)*^*LLL*^ gain Notch activity to a similar degree likely due to a lack of H-binding and repressor complex formation.

### Su(H) protein appears to be stabilised within the repressor complex

**Fig 4:**
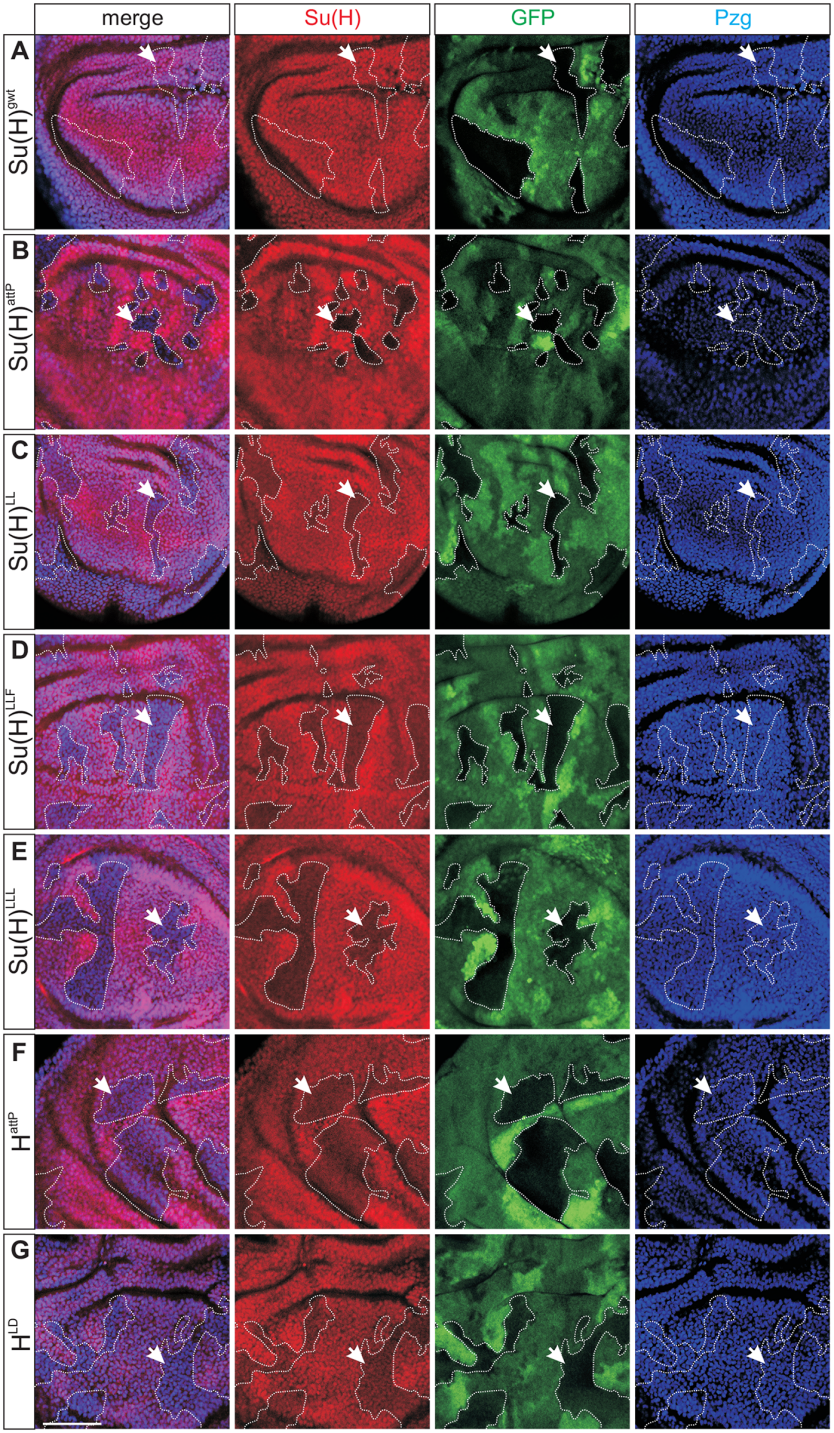
Abundance of Su(H) protein depends on its binding to Hairless. Clonal analysis was performed to induce homozygous mutant and wild type cells within the indicated heterozygous background in wing imaginal discs; the central wing blade is shown. Cells were stained for Su(H) (red), GFP (green) and Pzg (blue) protein. The latter serves as nuclear marker [64]. GFP marks wild type homo- and heterozygous cells; homozygous mutant cells lack GFP (outlined for clarity; arrows point to examples). The merge shows an overlay of Su(H) and Pzg. **(A)** Control clones of *Su(H)*^*gwt*^ reveal no changes in Su(H) protein accumulation (arrow). **(B)** As expected, cells homozygous for the null allele *Su(H)*^*attP*^ lack Su(H) protein (arrow). **(C-E)** Cell clones homozygous for either H-binding deficient mutant *Su(H)*^*LL*^ **(C)**, *Su(H)*^*LLF*^ **(D)**, or *Su(H)*^*LLL*^ **(E)** show a weaker and more fuzzy expression of the respective mutant Su(H) protein. **(F)** Cells homozygous for *H*^*attP*^ and hence devoid of H protein show a similar fuzzy expression of wild type Su(H) protein (arrow). **(G)** H^LD^ protein is defective in Su(H) protein binding. Cell clones homozygous for *H*^*LD*^ again display altered and reduced Su(H) protein accumulation (arrow). Size bar represents 50μm in all panels.

Curiously, despite its function as a transcriptional regulator Su(H) relies on NICD or H for nuclear import [28–32]. Our new *Su(H)* mutants allow to directly address how the lack of H binding might affect subcellular localisation of Su(H) protein. Based on previous work [29–32], we expected a shift from the nuclear to the cytoplasmic compartment. In cells homozygous for either *Su(H)*^*LL*^, *Su(H)*^*LLF*^, or *Su(H)*^*LLL*^, however, the Su(H) staining was remarkably weaker and appeared less distinct than in the neighbouring heterozygous or homozygous wild type cells, where Su(H) protein was concentrated within the nucleus (Fig 4A–4E). In the control *Su(H)*^*gwt*^ no differences between homo- or heterozygous cells were noted, whereas in *Su(H)*^*attP*^ mutant cells Su(H) signals were nearly absent as expected for a null mutant (Fig 4A and 4B). The reduced levels of mutant Su(H) protein suggested to us that Su(H) protein is stabilised while bound to H in the repressor complex (Fig 4C–4E). In this case, we would expect a likewise altered staining pattern of wild type Su(H) protein if H protein was absent or deficient for Su(H) binding. To this end, we generated cell clones homozygous for either *H*^*attP*^ or *H*^*LD*^ mutant—the former lack H protein, whereas the latter express a Su(H)-binding defective isoform of H [8,17]. As shown in Fig 4F and 4G, cells that are homozygous mutant for either *H* allele display a likewise weak and fuzzy Su(H) protein accumulation as seen for the H-binding deficient *Su(H)* mutants. To exclude an antibody-specific artefact, the experiments were repeated with a different polyclonal Su(H) antiserum that gave a similar result despite an overall higher background (S4 Fig). Signal quantification within the clones confirmed the visual impression: a reduction of cytoplasmic and more strongly of nuclear Su(H) signals resulted in a nearly equal subcellular distribution at very low levels (S7E Fig). An analysis of salivary glands from homozygous mutants corroborated these data: Su(H) protein was present at much lower level in nuclei, and not enriched in the cytoplasm in the absence of H binding (S5 Fig), demonstrating that we were not observing just a nuclear shuttling defect. Taken together, these data strongly suggest that the differences in Su(H) protein abundance depend upon interactions with H and are not an intrinsic property of the mutant Su(H) proteins *per se*.

Several feedback loops are built into the Notch signalling pathway [2,4]. To exclude that by manipulating the properties of Su(H) we may indirectly affect *Su(H)* transcriptional regulation, we quantified *Su(H)* mRNA expression in the H-binding deficient *Su(H)* alleles as well as in the *H* alleles by qPCR: as shown in supplemental S6 Fig expression levels were within the same range. Moreover, no changes in Su(H) protein expression were observed in cells homozygous mutant for *mam*^*2*^, a strong allele expected to disrupt the Notch signalling output [33] (S6 Fig). In sum we can exclude that the reduction of mutant Su(H) protein levels is a result of transcriptional down-regulation of the *Su(H)* gene.

**Fig 5:**
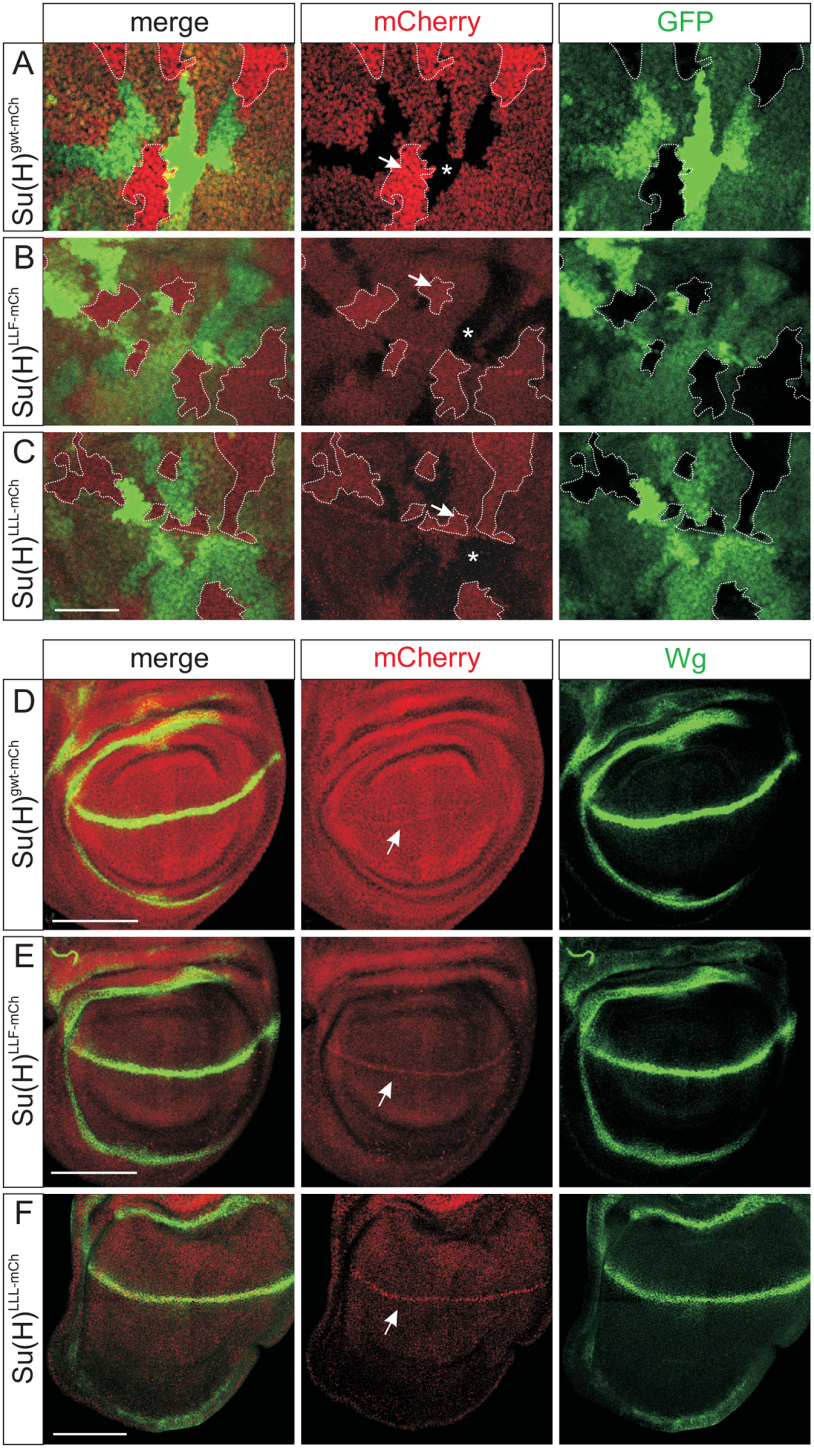
Lowered levels of Su(H)^LLF-mCh^ and Su(H)^LLL-mCh^ proteins. *Su(H)*^*gwt-mCh*^, *Su(H)*^*LLF-mCh*^, and *Su(H)*^*LLL-mCh*^ fly lines express mCherry-tagged versions of the respective proteins, which were detected with antibodies directed against mCherry. **(A-C)** Homozygous clones were induced in wing discs that are marked by the absence of GFP (green), and stained for mCherry tagged proteins (red). **(A)** Su(H)^gwt-mCh^ protein strongly accumulates in nuclei of homozygous cells (arrow) and is completely absent from the GFP labelled wild type sister cells (asterisk). Heterozygous cells display reduced levels and appear yellow in the merge. **(B, C)** In contrast, Su(H)^LLF-mCh^ or Su(H)^LLL-mCh^ protein accumulates at much lower levels compared to GFP, and is less distinct within nuclei. Intensity of the red channel was increased to visualize the staining. **(D-F)** mCherry (red) was detected in wing imaginal discs of homozygous animals. As read out for Notch activity, Wg protein is shown (green). Whereas *Su(H)*^*gwt-mCh*^ discs appear normal in shape as well as in Wg and Su(H) protein expression **(D)**, *Su(H)*^*LLF-mCh*^ and *Su(H)*^*LLL-mCh*^ mutant discs are hypertrophied **(E, F)** similar to the untagged version (compare with Fig 2E’ and 2F’). Moreover, Su(H)^LLF-mCh^ and Su(H)^LLL-mCh^ protein is strongly reduced overall, yet appears in a stripe where Notch activity is highest (arrow). Size bar represents 50μm in (A-C), and 100μm in (D-F).

As we were puzzled by the lowered signal intensity, we went ahead to directly follow Su(H) protein using mCherry as a protein tag [34]. Three further *Su(H)* fly lines were generated expressing the wild type, Su(H)^LLF^, and Su(H)^LLL^ proteins tagged with mCherry (S2B and S2E Fig). Using antibodies directed against mCherry, the results resembled those described above: the wild type Su(H)^gwt-mCh^ protein was conspicuously enriched within the nucleus, whereas Su(H)^LLF-mCh^ and Su(H)^LLL-mCh^ signals were weak and less distinct (Fig 5A–5C). Signal quantification within the clones revealed an about four-fold reduction of the Su(H)^LLF-mCh^ and Su(H)^LLL-mCh^ signals compared to that of Su(H)^gwt-mCh^ (S7 Fig). Again, drop of mutant protein levels were primarily seen in nuclei, for example of salivary glands (S5 Fig). Micrographs of endogenous fluorescence revealed the differences even more dramatically, as mutant Su(H) protein levels were barely above background in contrast to Su(H)^gwt-mCh^ (S7 Fig). H protein accumulation, however, was unchanged in *Su(H)* mutant cells (S8 Fig) [30]. These data emphasize that stability of Su(H) protein depends on the binding to H. Perhaps Su(H) protein is protected from degradation when present in the nucleus whilst assembled in the repressor complex. Hence, H may have an additional role with regard to Su(H) availability during Notch signalling processes, apart from its function as a co-repressor.

### Su(H) protein is stabilised by the binding to Hairless and to Notch

If we hypothesize that Su(H) is protected from degradation when nuclear and assembled within the repressor complex, it might likewise be protected within the activator complex when bound to NICD. Notch signalling is specifically activated along the dorso-ventral boundary of the wing imaginal disc [24,35]. Indeed, Su(H)^LLL-mCh^ and Su(H)^LLF-mCh^ proteins appeared specifically enriched in these cells (Fig 5D–5F).

**Fig 6:**
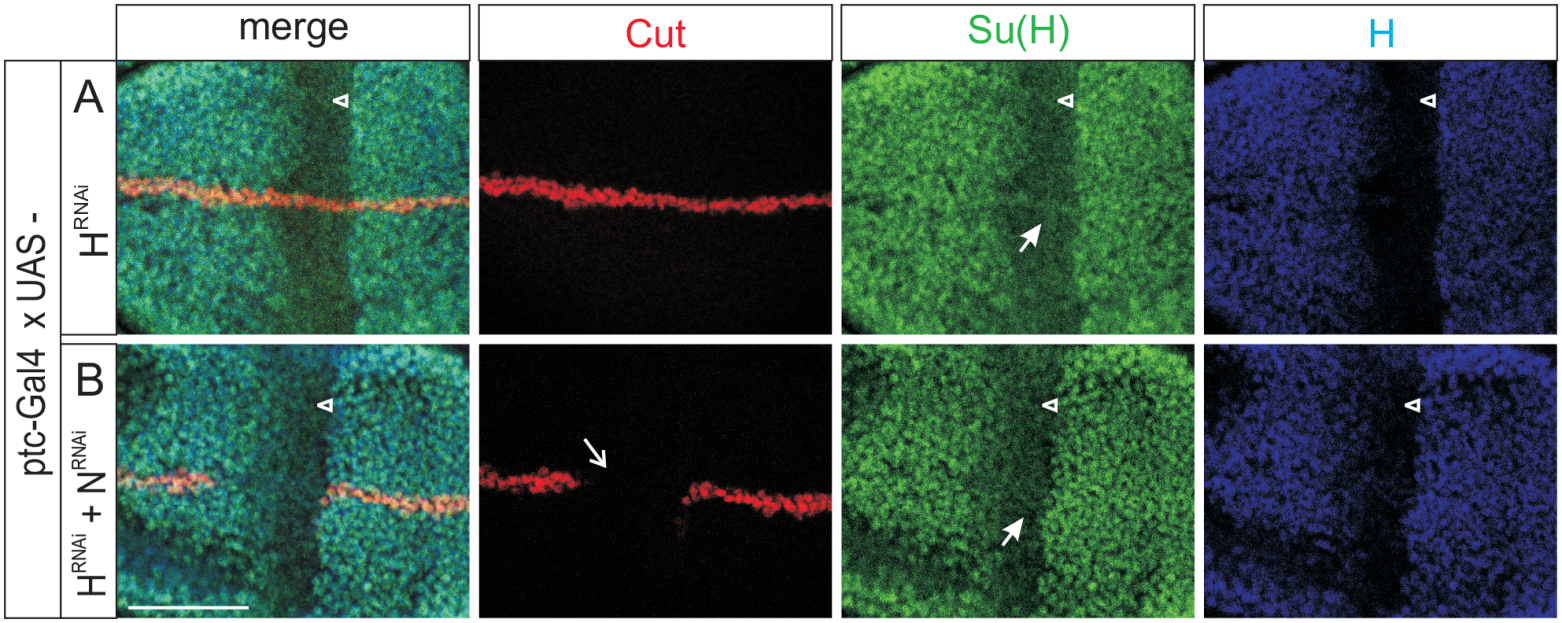
Accumulation of Su(H) mutant protein depends on both H and Notch. *H* and *Notch* activity was downregulated by tissue-specific RNAi along the antero-posterior boundary of wing imaginal discs by driving UAS-H^RNAi^ and/or UAS-N^RNAi^ with *ptc*-Gal4. Consequences on Su(H) (green) and H (blue) protein distribution, as well as on Cut expression (red) as a read out for Notch activity were investigated. **(A)** Su(H) protein accumulation is decreased specifically within the H-RNAi domain (arrowhead), whereas Cut expression appears normal. Note however, that Su(H) expression remains present along the dorso-ventral boundary (closed arrow). **(B)** Combined knock down of H and Notch activities gives the expected combination of a downregulation of Cut expression (open arrow) due to the lack of Notch activity, as well as of Su(H) expression (arrowhead) due to the lack of H. The specific Su(H) expression along the dorso-ventral boundary, however, is also lost in this genetic background (closed arrow), suggesting that Su(H) protein is stabilised by Notch in the absence of H [compare with (A)]. Size bar represents 50μm in all panels.

To further substantiate this idea, we turned to RNAi analyses. Knock down of H protein levels along the antero-posterior border of wing imaginal discs resulted in a strong decrease of Su(H) protein levels–yet, a weak signal was still present specifically along the dorso-ventral boundary (Fig 6A). As predicted by our model, the Su(H) boundary-accumulation was completely lost, when we knocked down Notch at the same time (Fig 6B), presumably because none of the complex forming proteins was present any longer.

### Stability of Su(H) protein in the activator complex

**Fig 7:**
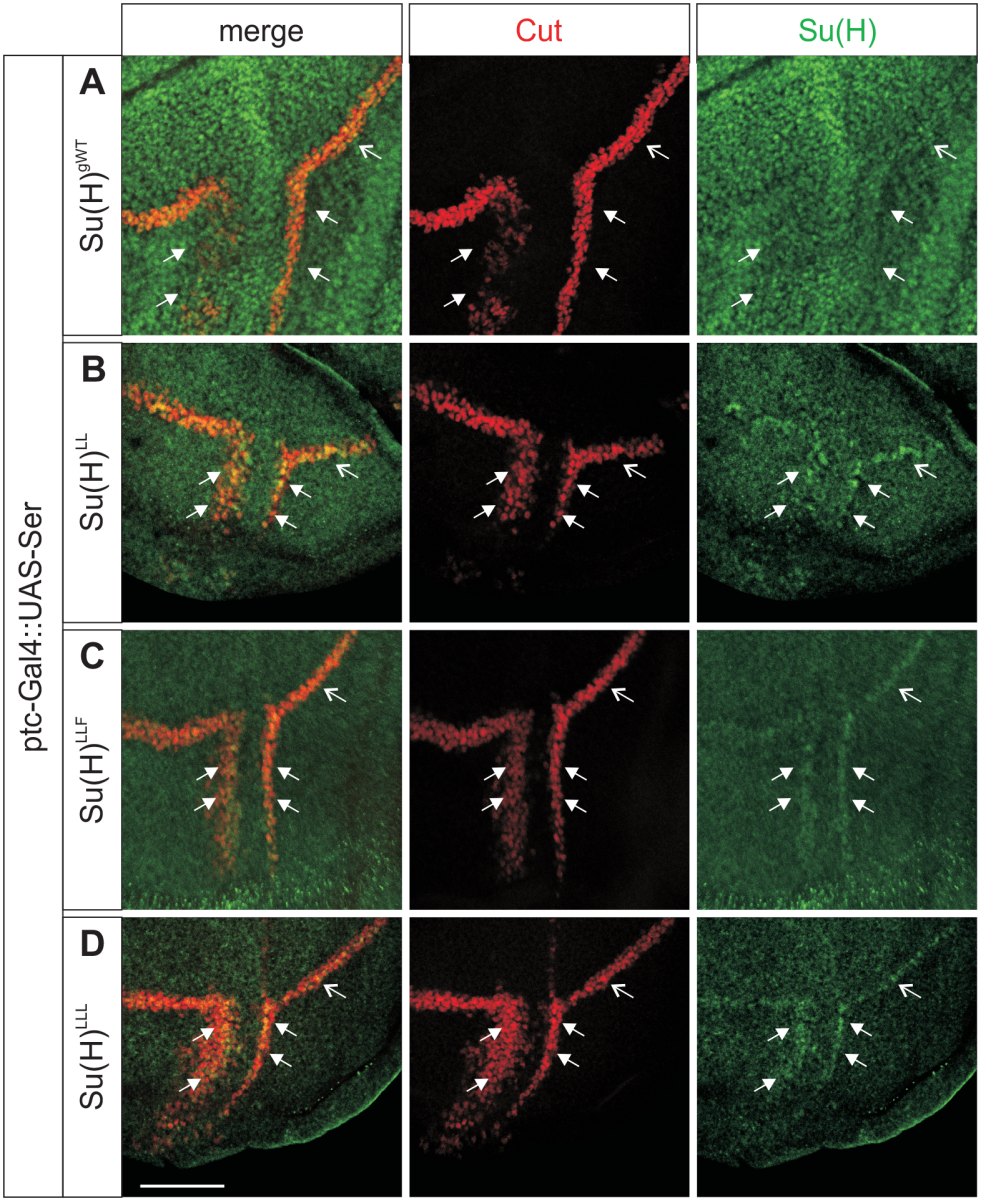
Su(H) mutant protein accumulates in response to Notch signal activation. Notch signalling was activated along the antero-posterior boundary within the ventral compartment of the wing disc by the overexpression of UAS-*Ser* with *ptc*-Gal4. Cut protein expression (red) served as read out. Su(H) protein is shown in green. **(A)** In a *Su(H)*^*gwt*^ background, Cut is ectopically induced in two stripes straddling the Ser-expression domain (closed arrows), in addition to its normal expression domain along the dorso-ventral boundary (open arrow). Su(H) protein is expressed at high levels overall. **(B-D)** The experiment was repeated in a homozygous mutant background of the H-binding deficient *Su(H)* mutants **(B)** *Su(H)*^*LL*^, **(C)** *Su(H)*^*LLF*^, or **(D)** *Su(H)*^*LLL*^. Su(H) protein levels are generally low, however, the mutant proteins accumulate specifically at the border of highest Notch activity (arrows). Size bar represents 50μm in all panels.

**Fig 8:**
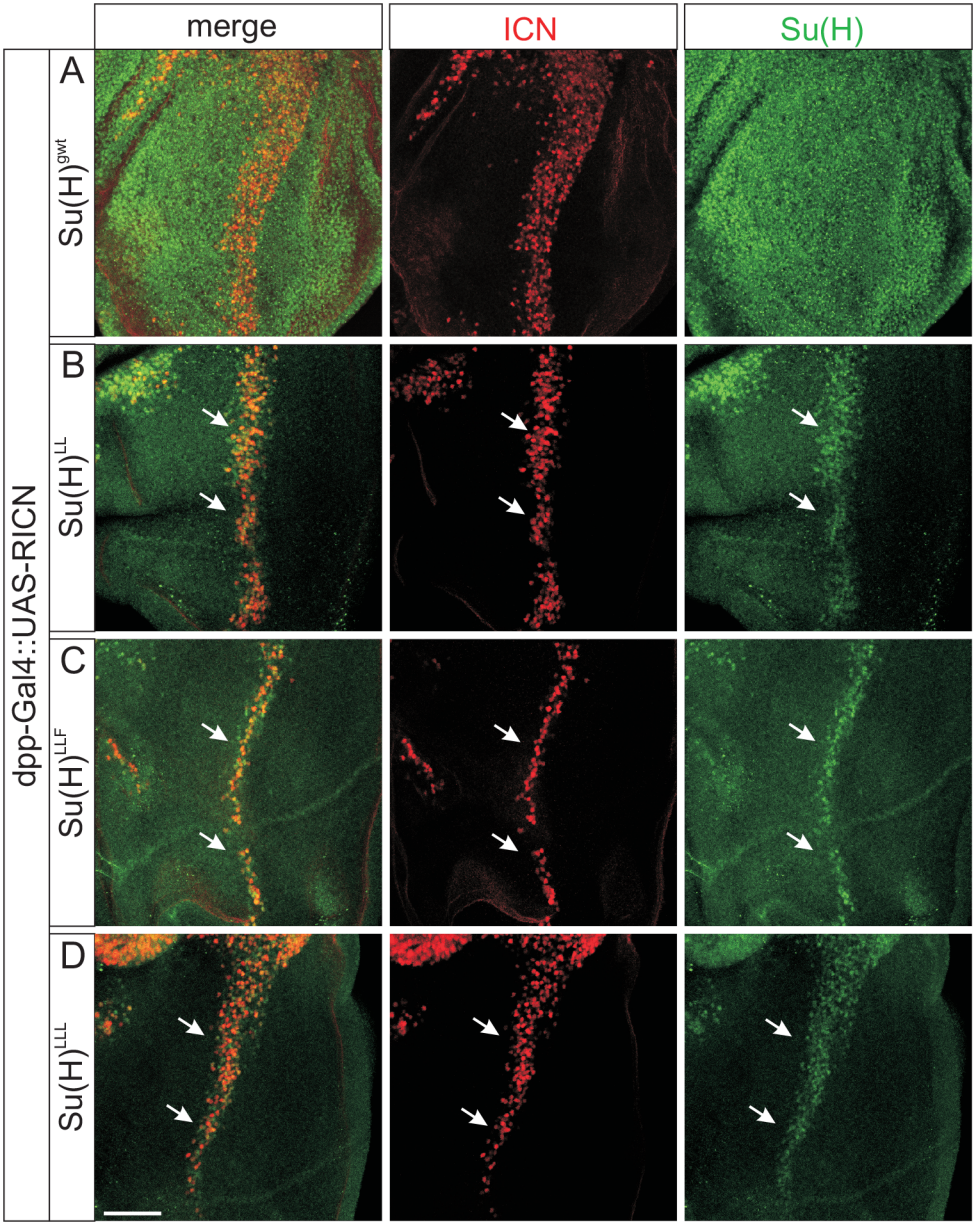
NICD overexpression results in Su(H) mutant protein accumulation. The Notch intracellular domain (UAS-RICN) was overexpressed along the dorso-ventral boundary of wing imaginal discs using *dpp*-Gal4. The animals were homozygous for the given *Su(H)* allele. Antibodies detect Notch intracellular domain (red) and Su(H) protein (green). **(A)** In the control, Su(H) protein appears largely unchanged due to high overall expression levels. H-binding deficient mutant proteins Su(H)^LL^ **(B)**, Su(H)^LLF^ **(C)**, or Su(H)^LLL^ **(D)**, in contrast, are each enriched within the domain of NICD overexpression (arrows). Size bar represents 50μm in all panels.

In order to test the influence of NICD on Su(H) protein accumulation, we specifically induced the pathway in an ectopic location by misexpressing the ligand Serrate (Ser) along the antero-posterior boundary in the wing imaginal disc. In this setting, the Notch signalling cascade is induced specifically within the ventral domain of the wing disc [35–38]. Moreover, cis-inhibition resolves the Notch response to two stripes straddling the ventral Ser expression domain [38,39], which was visualized by Cut protein expression as a read out (Fig 7). In a control disc little difference in Su(H) protein abundance was detected due to high endogenous levels (Fig 7A). In the homozygous mutant background of *Su(H)*^*LL*^, *Su(H)*^*LLF*^, or *Su(H)*^*LLL*^, however, where Su(H) protein levels are low, the signal enhancement was clearly seen at places of the strongest Notch activation, i.e. exactly along the border of Cut induction (Fig 7B–7D). Next we overexpressed NICD along the antero-posterior border of the mutant wing imaginal discs: indeed accumulation of the mutant Su(H) protein was observed demonstrating that NICD itself is sufficient for Su(H) protein stabilisation (Fig 8). These data strongly indicate that H-binding deficient Su(H) protein is bound and stabilised by NICD protein, supporting the notion of a stabilisation of Su(H) within the nucleus when assembled in the activator complex.

## Discussion

In this work, three H-binding deficient *Su(H)* alleles, *Su(H)*^*LL*^, *Su(H)*^*LLF*^, and *Su(H)*^*LLL*^, were generated by genome engineering. These mutations were based on our previous findings that alanine substitutions at these positions specifically affected repressor but not activator complex formation [11]. *In vivo*, we noted only little differences amongst the three mutant alleles. With regards to fly viability and SOP formation, however, *Su(H)*^*LL*^ appeared a somewhat weaker allele than the other two, in agreement with the residual H-binding activity this mutant displayed in yeast two-hybrid and overexpression assays [11]. All three alleles are larval to early pupal lethal, clearly demonstrating the pivotal role of Su(H) as a repressor during fly development. For example, maintenance of the SOP strictly depends on activity by the Su(H)-H repressor complex. In the case of low repressor activity, the SOP is prone to the epidermal fate through the presence of high levels of proneural proteins that activate *E(spl)* genes if not hindered [23]. Similar mechanisms likely occur in many other tissues, as *E(spl)* genes are Notch targets in almost every instance of Notch signalling, including the nervous system, the mesoderm, and the germ line (reviewed in [40]). Our expectation for the H-binding deficient *Su(H)* alleles, namely gain of Notch activity, was confirmed by our data. In fact, the three alleles were nearly indistinguishable from a complete loss of *H* in homozygosis. This allows the conclusion that the mutant Su(H) proteins indeed fail to form a repressor complex *in vivo*. Moreover, these results show that the primary function of H during imaginal development is repression of Notch signalling. During embryogenesis, however, H may take part in additional repressor activities [41]. Originally Su(H) was identified as a dominant suppressor of the *H* heterozygous bristle loss phenotype [42,43]. Positive and negative autoregulation of *Su(H)* in the context of mechano-sensory organ formation in addition to several feedback loops built into the Notch signalling pathway, however, complicate genetic analyses [2,4,13,28, 42–45]

Importantly, our studies revealed a novel additional regulatory mechanism of Notch signal transduction at the level of Su(H) protein stability. Our data demonstrate that Su(H) protein is stabilised when in complexes with either H or NICD, but appears to be unstable when unbound. Accordingly, mutant cells lacking H protein or expressing H protein deficient in Su(H) binding show low Su(H) protein levels (Figs 4–6). Likewise H-binding deficient Su(H) protein is barely detected apart from regions of highest Notch activity (Figs 6–8). Apparently, Su(H) protein levels are ruled by the amount of H or NICD within a cell, consistent with the normal appearance of the heterozygotes. Whereas H protein is unaffected by Su(H) levels (S8 Fig) [30], there are indications for a mutual inter-dependence of overall Notch and Su(H) protein levels during *Drosophila* embryogenesis [46]. Taken together, our data now implicate a novel role for H in the regulation of Su(H) availability apart from its role as co-repressor. The mouse homologue RBPJ is a rather unstable protein with a half life of about 2 hours [47]. RBPJ degradation involves both the proteasome and the lysosome and is regulated by p38 MAPK phosphorylation and Presenilin-2 [47]. Possibly, Su(H) protein is protected from degradation as long as it is bound within protein complexes, be it activator or repressor complexes. It remains to be established, whether NICD or co-repressors protect RBPJ from degradation similar to Su(H). As both H and NICD are involved in the nuclear shuttling of Su(H), stability may likewise (or in addition) depend on subcellular localisation. To date, we cannot distinguish between these two aspects, as this requires further and more detailed analyses of Su(H) stability. In mammals, however, CBF1/RBPJ nuclear translocation has been shown to also depend on the binding of either co-repressors or NICD [48].

Based on their Rel homology region domains (RHR), CSL proteins are considered distant relatives of the rel class of transcription factors [49]. Our data now add new details to this relationship. The immunoglobulin-like folds, corresponding to RHR-c, build the centre of the CSL C-terminal domain (CTD) that contacts both NICD and MAM in the activator complex [50,51], as well as H in the repressor complex [8, 11]. The homologous structure in NFκB for example binds to the inhibitory protein IκB resulting in cytoplasmic retention of NFκB (reviewed in [52,53]). Similar to NICD, IκB contains six or more Ank-repeats that contact RHR-c, or CTD in CSL (reviewed in [52]). In general, rel proteins become activated through their nuclear translocation, which is inhibited by IκB proteins. The signalling cascade eventually activating rel proteins results in a phosphorylation and degradation of IκB, and subsequent nuclear translocation of NFκB (reviewed in [53]). Clearly, CSL proteins differ in several respects. Firstly, they act as monomers unlike the rel type transcription factors [5]. Secondly, their nuclear import requires a co-factor rather than the release from an inhibitor. In both examples, however, regulation of nucleo-cytoplasmic shuttling and degradation are important steps in the process of signal transduction.

## Materials and methods

### Generation of the *Su(H)*^*attP*^ founder line

Genome engineering was performed as outlined before [16,17]. Genomic fragments were derived from two overlapping λ phages isolated from the *Kr*^*Sb10*^/SM1 library [54] and cloned into pGX-attP [16]: into the *Kpn* I site of the 5’MSC a 3.4 kb *Eco* RI fragment and into the *Bgl* II / *Xho* I sites of the 3’MSC a 3.9 kb *Bam* HI / *Xho* I fragment. The construct was inserted randomly into the genome in the HR-starter line by P-element mediated germ line transformation [55]. Homologous recombination at the *Su(H)* locus was induced after crossing in *y*^*1*^ *w*^*1118*^; P{ry*, 70Flp}11P{*v*+,70I-SceI}2B *Sco*/CyO (BL6934) and subjecting the offspring to a heat shock regimen as described before [56]. One recombinant event was isolated from about 850 mosaic F1 virgins. The founder line *Su(H)*^*attP*^ was derived by *Cre*-mediated deletion of the *white*^+^ marker and vector-remains by crossing in *y*^*1*^ *w*^*67c23*^; *sna*^*Sco*^ / CyO, P{w[+mC] = Crew}DH1 (BL1092) as described before [17,56]. *Su(H)*^*attP*^ carries a deletion of 1.75 kb of genomic DNA and contains an attP site instead. The line was verified by PCR and sequence analysis.

### Generation of Su(H) constructs and *Su(H)* mutant fly lines

The genomic insertion construct was derived from the already mentioned λ phage clone and contains a 2.3 kb *Eco* RV / *Nde* I fragment; it was cloned as *Bam* HI / *Xho* I fragment into pGE-attB^GMR^ to be inserted into the attP site of the founder line as outlined before [16,17]. After floxing the *white*^+^ and vector sequences, the resultant *Su(H)*^*gwt*^ line contains a duplication of 590 bp of intronic sequences plus remaining attR, loxP, and vector sequences in the intron and the 3’ UTR. This strategy was chosen to avoid possible splice defects. The three alanine substitution mutations were originally introduced in *Su(H)* cDNA [11]. They were shuttled into the genomic *Su(H)* DNA whenever possible, resulting in the loss of some of the three introns: *Su(H)*^*LL*^ and *Su(H)*^*LLF*^ lack intron 3, and *Su(H)*^*LLL*^ lacks introns 2 and 3. The respective control lines *Su(H)*^*gwtΔi3*^ and *Su(H)*^*gwtΔi2i3*^ were generated as well. In addition, *Su(H)*^*gwt-mCh*^, *Su(H)*^*LLF-mCh*^ and *Su(H)*^*LLL-mCh*^ were produced by C-terminal in frame fusion of mCherry that was derived from pRRins [34]. Mutant and control constructs were inserted into the attP site of the founder line by site specific recombination and *white*^+^ plus vector sequences were deleted as described above. The integration frequency varied between 2–7% of larvae surviving the injection. All lines were confirmed by PCR, diagnostic restriction digests and sequence analysis.

### Fly work

Flies were raised on standard fly food at 18°C; crosses were kept at 25°C. The following stocks were used: Oregon R, *y*^*1*^ *w*^*67c23*^ (BL6599), *H*^*attP*^/TM6B [17], *H*^*LD*^/TM6B [17], *Su(H)*^*Δ47*^/CyO [57], *Su(H)*^*SF8*^/CyO [43]; *ptc*-Gal4 (BL2017) [58], *dpp*-Gal4 [59], UAS-*Ser* (BL5815) [36], UAS-RICN (corresponding to UAS-NICD) [32], UAS-H^RNAi^ [14], UAS-N^RNAi^ (BL7078) [60]. Details on fly strains are found in Flybase (flybase.org). Stocks were combined or recombined by standard genetics; genotypes were verified by PCR and diagnostic restriction digests. *Su(H)* mutants were balanced over CyO-GFP to allow selection of homozygous larvae based the GFP fluorescence, using a Leica UV-dissecting microscope MZ FL III with a GFP filter set. *H* mutants were balanced over TM6B, and the homozygotes selected by the absence of the *Tubby* marker. Mutant phenotypes were recorded as described before [17].

Notch activity was induced along the antero-posterior boundary by crossing UAS-*Ser* with *ptc*-Gal4 as described earlier [36], or by crossing UAS-RICN [32] with *dpp*-Gal4 [59] in a wild type or *Su(H)* mutant background. Mosaics were induced with the FLP/FRT technique [61,62]; to this end FRT40A was recombined with the *Su(H)* alleles of interest and crossed with *y*^*1*^ w*; P{w^+mC^ = Ubi-GFP.D}33 P{w^+mC^ = Ubi-GFP.D}38 P{*ry*^*+t7*.*2*^neo-FRT)40A / CyO (BL5189). Offspring was heat shocked for 1h at 37°C, at first to second instar larval stage and dissected as wandering third instar larvae. Scanning electron micrographs were taken from adult female flies using a table-top NeoScope (JCM-5000; Nikon, Tokyo, Japan).

### Immunochemistry

For Western blots, about 10 homozygous third instar larvae were homogenized in 100μl binding buffer (20mM HEPES pH 7.6, 150mM KCl, 2.5mM MgCl_2_, 10% glycerol, 0.05% NP-40, 1mM DTT, ROCHE complete ULTRA protease inhibitor mini tablet) [63], and protein amounts were adjusted by larval weight and normalized by Bradford assay. Rabbit anti-Su(H) (1:1000; Santa Cruz Biotech, Dallas, USA) or mouse anti-beta-tubulin A7 (1:3000) (developed by M. Klymkowsky; obtained from DSHB, the Developmental Studies Hybridoma Bank developed under the auspices of the NICHD and maintained by the University of Iowa, Dept. of Biology, Iowa City, IA 52242) and goat anti-rabbit or anti-mouse coupled to alkaline phophatase (from Jackson Immuno-Research, via Dianova, Hamburg, Germany) were used for detection. The blots were cut before separate detection of Su(H) and beta-tubulin proteins.

Wing imaginal discs from late third instar larvae were dissected and stained according to standard protocols. The following antibodies were used: rabbit anti-Su(H) (1:1000) in all experiments except for S4 Fig and rabbit or mouse anti-GFP (1:50) (all from Santa Cruz Biotech, Dallas, USA), rabbit anti-mCherry (1:500) (Gene Tex, Irvine, USA), rat anti-Su(H) (1:200) [32] in S4 Fig, guinea pig anti-Hairless A (1:500) [30], guinea pig anti-Pzg (1:500) [64], mouse anti-Cut 2B10 (1:25), anti-Hnt 1G9 (1:10), anti-Wg 4D4 (1:25) and anti-Notch intracellular domain C17.9C6 (1:50) (developed by G. Rubin, H.D. Lipshitz, S.M. Cohen, and S. Artavanis-Tsakonas respectively; obtained from DSHB). Goat or donkey secondary antibodies with minimal cross-reactivity, coupled to FITC, Cy3, or Cy5 were obtained from Jackson Immuno-Research (Dianova, Hamburg, Germany). Tissue was mounted in Vectashield (Vector labs, Eching, Germany), examined with Zeiss Axioskop coupled to a BioRad MRC1024 confocal microscope using LaserSharp 2000 software (Carl Zeiss, Jena, Germany). Pictures were assembled using *Image J*, *Photo Paint* and *Corel Draw* software.

### RT-PCR and RT-qPCR

Poly(A)^+^ RNA was extracted from 100mg of female flies from either Oregon R or *Su(H)*^*gwt*^ using the Poly ATract 1000 kit (Promega, Mannheim, Germany), and cDNA generated with ProtoScriptII First Strand cDNA Synthesis Kit (New England Biolabs, Frankfurt, Germany) according to the suppliers’ protocols. The PCR spans the first intron that contains the manipulations: Genomic DNA from wild type and from *Su(H)*^*gwt*^ should yield a 1012 bp and a 1745 bp amplificate, respectively. The amplificate from cDNA is expected to be 238 bp in size when spliced normally. The following primer pair was used: Pu, 5’ CCG GCC ACA CAT CGA GGA GAA G 3’ and Pl, 5’ CGC GCA TAG TTG TGC TCC CTG TTC G 3’.

qPCR was done on three biological replicates of each genotype with 40 homozygous larvae at ~72 hours after egg deposition at 25°C. Poly(A)^+^ RNA was extracted with Poly ATract^®^ 1000 kit (Promega, Mannheim, Germany) and the concentration determined in a μCuvette with the BioPhotometer Plus (Eppendorf, Hamburg, Germany). 1μg was treated with 0.4U DNase I (New England Biolabs, Frankfurt, Germany) and reverse transcribed in 0.3μg batches with the ProtoScriptII Kit using oligo-dT primers (New England Biolabs, Frankfurt, Germany). Real time qPCR was performed with Blue S’Green qPCR Kit (Biozym, Hessisch-Oldendorf, Germany) on 2μl of cDNA (0.012μg) in 10μl end volume using MIC magnetic induction cycler (bms, Pots Point Australia) always including target and no-template controls; a hot start (95°C 2 min) and 40 cycles of 95°C 5s / 68°C 10s was followed by a melt curve analysis (72°C to 95°C at 0.3°/s) to select for specific amplification. Absence of DNA was tested in a non-RT control for every sample; RNA integrity was confirmed by 5’-3’ Cq analysis. *CTCF* (PP30808), *cyp33* (PP14577), *DNApol-α60* (PP9936), *eRF1* (PP11596), *hisRS* (PP13550), and *Tbp* (PP1556) were assayed as internal references; primer pair sequences (in parentheses) are listed at DRSC FlyPrimer bank [65]. *Su(H)* primers (Upper, 5’ CAT ATC CAC CGA CAA GGC TGA GTA CC 3’; Lower, 5’ TAA CGA TTG GCA CTG GAG TGA CTG G 3’) span the second intron. Eventually, *cyp33* and *Tbp* were selected based on variance, Cq values, and expression profiles matching that of *Su(H)* (DRSC FlyPrimer bank). Relative quantification of the data was performed with *micPCR* software Version 2.2 based on *REST* [66], taking target efficiency into account.

## Supporting information

S1 Fig Procedure of genome engineering. **(A)** The scheme depicts the structure of *Su(H)*^*attP[w+]*^, established by homologous recombination. **(B)** The *white*^+^ marker and flanking sequences were floxed out by Cre-mediated recombination, resulting in *Su(H)*^*attP*^. This founder line served as origin for the integration of any Su(H)* DNA construct cloned into pGE-attB^GMR^, as outlined for the genomic rescue construct below. **(C)** The genomic *Su(H)*^*gwt*^ rescue construct was inserted as a proof of principle, giving *Su(H)*^*gwt [w+]*^. **(D)** The final control line *Su(H)*^*gwt*^ was established by deleting the *white*^+^ marker. The same procedure was applied for any other construct as well. Primer pair used for RT-PCR in (E) is not to scale. **(E)** Splicing of *Su(H)* mRNA occurs normally in the rescue strain *Su(H)*^*gwt*^ compared to the wild type strain Oregon R. RT-PCR was performed on cDNA from the respective strains, using primer pair shown in (D), to obtain the expected 238 bp fragment (arrowhead). Unlike the control (c), the probe (p) contained reverse transcriptase. Size standard (M) was a 100 bp ladder; some bands are labelled for clarity (* unspecific products). **(F)** Quantification of Western blots (n = 5) was performed with *Image J* gel analysis program. Beta-tubulin signals served as internal standard; mutants were compared to *Su(H)*^*gwt*^ control. Error bars represent standard deviation. Significance was tested by ANOVA two-tailed Tukey-Kramer approach for multiple comparisons (***, p<0.001).
(TIF)

S2 Fig Verification of the newly generated *Su(H)* alleles. **(A)** Structure of the *Su(H)* primary transcript. Dashed lines indicate positions of deletion and reintegration into the founder line *Su(H)*^*attP*^. Relevant restriction sites used for cloning are indicated. **(B)** Scheme of the control and mutant Su(H) constructs used for the generation of the respective *Su(H)* alleles. Note the absence of intron 3 in *Su(H)*^*LL*^ and *Su(H)*^*LLF*^, and of both introns 2 and 3 in *Su(H)*^*LLL*^. **(C)** Viability of the new *Su(H)* alleles balanced over CyO was assessed by a cross to *y*^*1*^ *w*^*67c23*^ control in comparison to *Su(H)*^*Δ47*^ and *Su(H)*^*SF8*^ alleles. The expected 1:1 ratio of mutant and balancer chromosome CyO was observed, yet *Su(H)*^*LLL*^ has a slightly lowered viability. **(D)** The new *Su(H)* alleles were crossed with *Su(H)*^*attP*^/CyO-GFP and the number of hemizygous pupae counted relative to the heterozygotes recognized by green fluorencence. *Su(H)*^*Δ47*^ and *Su(H)*^*SF8*^ served for comparison. **(E)** The homozygous control lines *Su(H)*^*gwt*^, *Su(H)*^*gwtΔi3*^, or *Su(H)*^*gwtΔi2i3*^ are like wild type, exemplified by a normal Wg staining. All three are able to complement the *Su(H)*^*attP*^ allele: they are viable and have normal looking wing discs in hemizygosis. Likewise, *Su(H)*^*gwt*-*mCh*^ appears wild type, whereas *Su(H)*^*LLL-mCh*^ and *Su(H)*^*LLF-mCh*^ display hypertrophied wing discs similar to the untagged strains (compare with Fig 2E’ and 2F’). Size bar represents 100μm in all panels. **(F)** Survival rates of the hemizygous controls *Su(H)*^*gwt*^, *Su(H)*^*gwtΔi3*^, or *Su(H)*^*gwtΔi2i3*^ over *Su(H)*^*attP*^ was determined relative to wild type stock Oregon R (OreR). Inset numbers in (C), (D), (F) represents animals analysed.
(TIF)

S3 Fig Wg expression in cell clones mutant for the new *Su(H)* alleles. Expression of Wg (red) was analysed in cell clones homozygous mutant for the allele indicated. Mutant clones are within the central wing blade; they are marked by the absence of GFP (green) and are outlined. Examples of altered Wg expression are highlighted by arrows. **(A)** Wg is expressed along the dorso-ventral boundary of the disc. Control clones of *Su(H)*^*gwt*^ do not disturb the wild type pattern. **(B)** Wg protein expression is lost from *Su(H)*^*att*P^ homozygous cells (arrow) due to the absence of Su(H) protein. **(C-E)** A slight broadening of Wg expression is seen in cell clones homozygous mutant for any of the H-binding deficient *Su(H)* alleles, *Su(H)*^*LL*^ **(C)**, *Su(H)*^*LLF*^ **(D)**, or *Su(H)*^*LLL*^ **(E)**. **(F-G)** A similar broadening is observed in cells lacking H activity (arrow), as in *H*^*attP*^ **(F)** or *H*^*LD*^ (G). Size bar represents 25μm in all panels.
(TIF)

S4 Fig Control clonal analysis of Su(H) protein accumulation. Clones were induced in wing imaginal discs; magnification of the central wing blade is shown. Genotypes are indicated on the left. Cells were stained with a polyclonal rat anti-Su(H) antiserum (red) [32], anti-GFP (green) as clonal, and anti-Pzg (blue) as nuclear marker [64]. Wild type homo- and heterozygous cells are marked by GFP; homozygous mutant cells lack GFP and are outlined for clarity (arrows point to examples). The merge shows an overlay of Su(H) and Pzg. **(A)** In contrast to *Su(H)*^*gwt*^ control clones, **(B)** *Su(H)*^*attP*^ null mutant cells show little Su(H) protein signals. **(C-E)** Note the reduction of Su(H) protein levels in H-binding deficient *Su(H)* mutant cells, **(F,G)** and alike in cells lacking H activity. (Bright dots in C,F,G are unspecific labelling). Size bar represents 50μm in all panels.
(TIF)

S5 Fig Su(H) protein expression in salivary glands. **(A, B)** Salivary glands from homozygous third instar larvae of the given genotype were doubly stained for Pzg as nuclear marker and for Su(H) and mCherry, respectively. Drastic reduction of nuclear Su(H) protein expression is apparent in the mutants. **(C)** Quantification of staining intensity was performed on stacks of pictures cutting through the entire gland using *Image J*. Intensity of all the nuclei from one gland was compared with the total cytoplasmic signal from that gland (n = 3–5). Loss of nuclear staining and no concurrent cytoplasmic enrichment is observed. **(D)** Signal intensity of Su(H) and Pzg staining of individual nuclei (25–50 per gland; 3–5 glands per genotype) were recorded using *Image J*. A four- to five-fold drop in Su(H) expression is seen in the H-binding deficient *Su(H)* as well as in the *H* alleles indicated. Differences between mutants and control are highly significant by ANOVA two-tailed Tukey-Kramer approach for multiple comparisons (***, p<0.001). Size bar represents 100μm in all panels.
(TIF)

S6 Fig Reduction of Su(H) protein levels is not a result of altered transcriptional regulation. **(A)** *Su(H)* mRNA expression was quantified by qPCR relative to *cyp33* and *Tbp* as reference genes. Efficiencies for *Su(H)* (0.97), for *cyp33* (0.94) and for *Tbp* (0.92) were taken into account for the relative quantification [65]. mRNA was derived from homozygous mutant larvae and compared to *Su(H)*^*gwt*^. Data are assembled from three biological and two to four technical replicates. Mini-max depicts 95% confidence, median corresponds to expression ratio. Values are close to 1, i.e. close to *Su(H)*^*gwt*^ expression; differences of about 20% between *Su(H)*^*gwt*^ and *H*^*attP*^, or *Su(H)*^*gwt*^ and *Su(H)*^*LLL*^ are assessed statistically significant, however, might be attributed to stage variations. **(B)** Cell clones homozygous mutant for *mam*^*2*^, distinguished by the lack of GFP (green), show no changes in Su(H) protein expression (red). Arrow points to an example. Size bar represents 100μm.
(TIF)

S7 Fig Endogenous signals of mCherry tagged Su(H) protein. **(A-C)** Clonal analysis in *Su(H)*^*gwt-mCh*^, *Su(H)*^*LLF-mCh*^, and *Su(H)*^*LLL-mCh*^ wing discs: red auto-fluorescence of mCherry is shown. Wild type cells are marked by GFP, displaying green auto-fluorescent GFP signals. Heterozygous cells show a weaker fluorescence signal of both. Clones are outlined for clarity. Size bar is 50μm. **(A)** The fluorescence signals of both the homozygous and heterozygous cells are clearly visible in *Su(H)*^*gwt-mCh*^ clones. **(B,C)** In contrast, mCherry signals in either *Su(H)*^*LLF-mCh*^ or *Su(H)*^*LLL-mCh*^ homozygous cells are just above background, precluding signal quantification. Intensity of the red channel was increased to visualize the staining. **(D)** Signal quantification of *Su(H)*^*gwt-mCh*^, *Su(H)*^*LLF-mCh*^, and *Su(H)*^*LLL-mCh*^ in clones stained with anti-mCherry and anti-GFP antibodies as in Fig 5A–5C. Signals were recorded at 20 points each within homozygous wildtype, mutant, and heterozygous clones at identical positions for mCherry and for GFP within each disc. To allow for comparison of intensity between control and mutant, homozygous (hz) or heterozygous (htz) mCherry signals were set in relation to GFP signals from heterozygous (htz) cells. Su(H)^gwt-mCh^ protein is about four-fold enriched compared to Su(H)^LLF-mCh^ or Su(H)^LLL-mCh^ protein. Signal intensity of mCherry correlates well with gene dose (heterozygous is about half of homozygous, htz/hz), in contrast to the GFP signals, where the heterozygous signal is more than two-fold weaker than the homozygous signal. Error bars indicate standard deviation (n = 5–7 biological samples). **(E)** Quantification of nuclear versus cytoplasmic Su(H) protein detected with rabbit anti-Su(H) antibodies in clones as shown in Fig 4. Signals were recorded within and just next to 20 nuclei each within homozygous wild type (dark columns) or homozygous mutant clone (light columns) at identical positions within each disc. GFP signals were used to identify cell clones and Pzg signals to identify the nuclei. Grey values were determined with *Image J*, and were related to the nuclear signal of the control. Red columns represent nuclear, grey columns cytoplasmic signals; dark columns represent homozygous wild type and light columns homozygous mutant. Error bars show standard deviation (n = 2–4 biological samples). In wild type cells Su(H) is approximately 1.2–1.4 fold enriched in the nucleus (upper numbers). No such enrichment is observed in any of the *Su(H)* binding deficient mutant cells, and likewise the *H* mutant cells (lower numbers). Moreover, only about 50% of signal intensity of homozygous wild type cells is reached in mutant cells. In homozygous *Su(H)*^*attP*^ cells signal intensity is about 25% of wild type, revealing the background level obtained with the polyclonal antibodies used in this study.
(TIF)

S8 Fig Hairless protein accumulation is independent of Su(H). (**A-B**) Clones homozygous for *Su(H)*^*gwt-mCh*^ **(A)** and *Su(H)*^*LLL-mCh*^ **(B)** were induced in wing imaginal discs and stained for mCherry (red) and for H protein (blue). (**C**) Likewise *Su(H)*^*attP*^ homozygous mutant cells lacking Su(H) protein were generated, and Su(H) protein (red) and H protein (blue) detected. Note equal distribution of H protein in all genotypes. Clones are marked with GFP [green in (A-C)]; homozygous wild type cells show high GFP levels, mutant cells lack GFP. The clones are outlined for clarity. Size bar represents 50μm in all panels.
(TIF)
