# Supplementary figures and images for: Hairless-binding deficient Suppressor of Hairless alleles reveal Su(H) protein levels are dependent on complex formation with Hairless
Source: PLoS Genet. 2017 May 5;13(5):e1006774. doi: 10.1371/journal.pgen.1006774 (PMC5438185; doi:10.1371/journal.pgen.1006774)

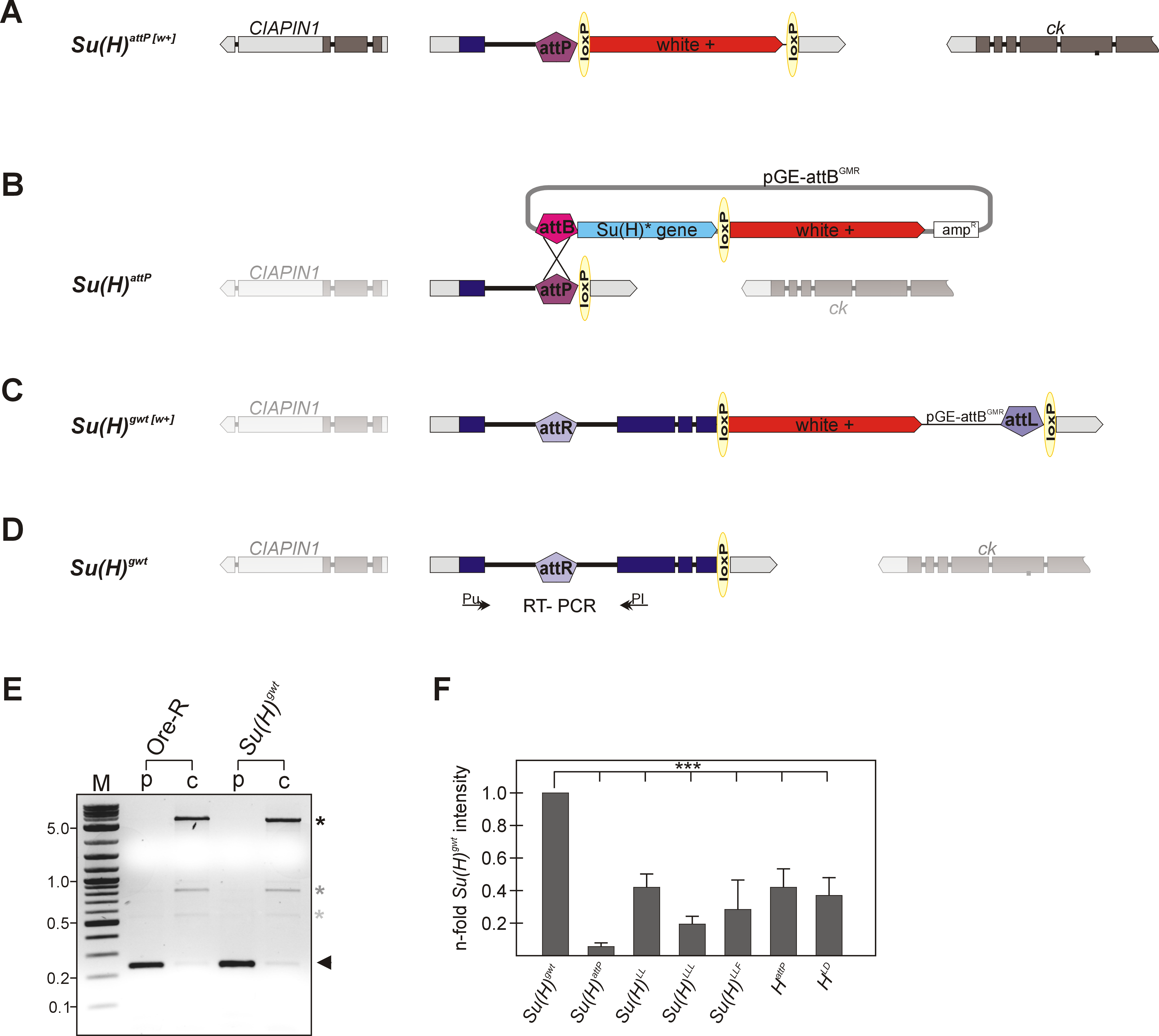

Supplement: S1 Fig — (A) The scheme depicts the structure of Su(H)attP[w+], established by homologous recombination. (B) The white+ marker and flanking sequences were floxed out by Cre-mediated recombination, resulting in Su(H)attP. This founder line served as origin for the integration of any Su(H)* DNA construct cloned into pGE-attBGMR, as outlined for the genomic rescue construct below. (C) The genomic Su(H)gwt rescue construct was inserted as a proof of principle, giving Su(H)gwt [w+]. (D) The final control line Su(H)gwt was established by deleting the white+ marker. The same procedure was applied for any other construct as well. Primer pair used for RT-PCR in (E) is not to scale. (E) Splicing of Su(H) mRNA occurs normally in the rescue strain Su(H)gwt compared to the wild type strain Oregon R. RT-PCR was performed on cDNA from the respective strains, using primer pair shown in (D), to obtain the expected 238 bp fragment (arrowhead). Unlike the control (c), the probe (p) contained reverse transcriptase. Size standard (M) was a 100 bp ladder; some bands are labelled for clarity (* unspecific products). (F) Quantification of Western blots (n = 5) was performed with Image J gel analysis program. Beta-tubulin signals served as internal standard; mutants were compared to Su(H)gwt control. Error bars represent standard deviation. Significance was tested by ANOVA two-tailed Tukey-Kramer approach for multiple comparisons (***, p<0.001). (TIF) [file pgen.1006774.s001.TIF]

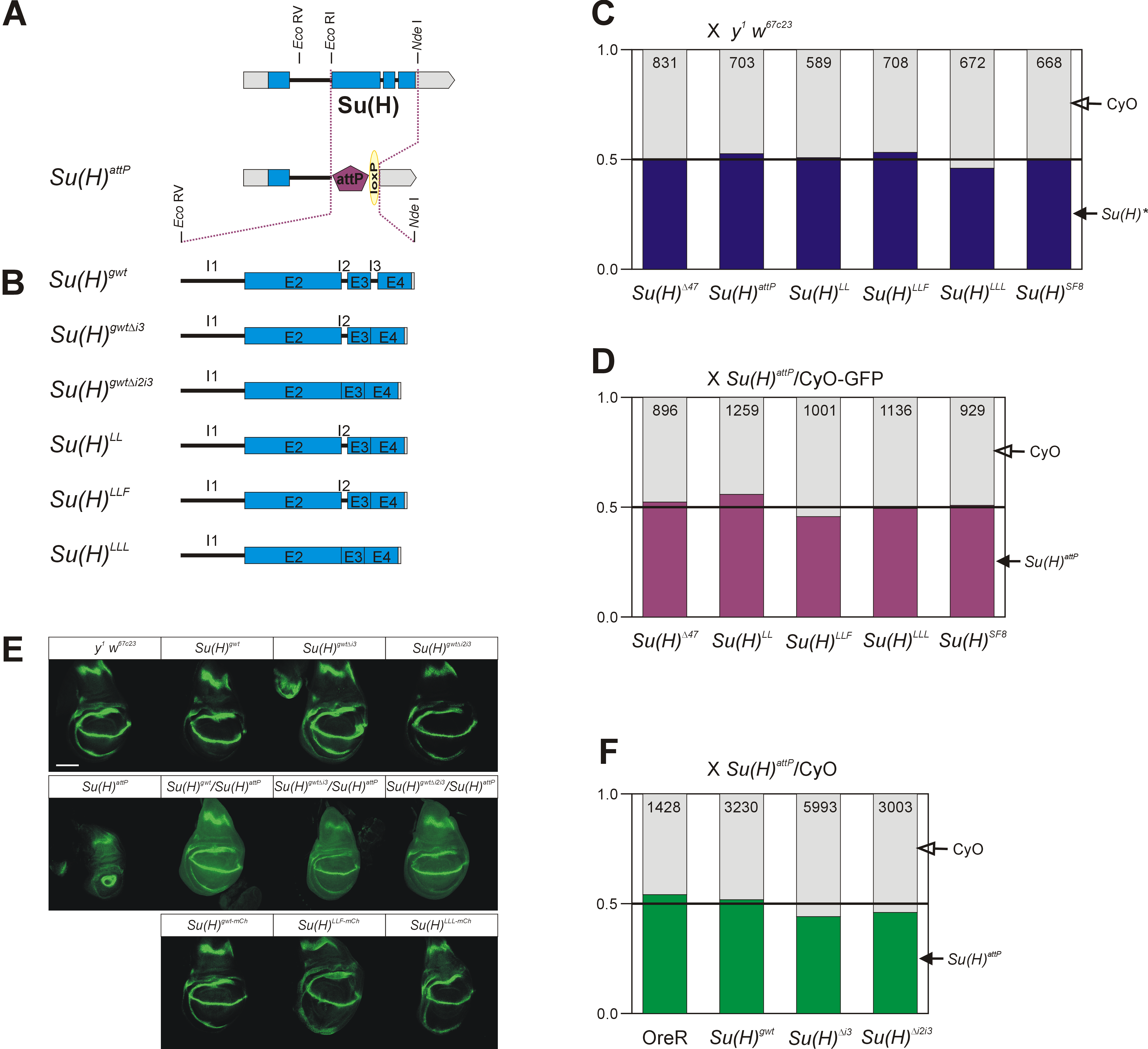

Supplement: S2 Fig — (A) Structure of the Su(H) primary transcript. Dashed lines indicate positions of deletion and reintegration into the founder line Su(H)attP. Relevant restriction sites used for cloning are indicated. (B) Scheme of the control and mutant Su(H) constructs used for the generation of the respective Su(H) alleles. Note the absence of intron 3 in Su(H)LL and Su(H)LLF, and of both introns 2 and 3 in Su(H)LLL. (C) Viability of the new Su(H) alleles balanced over CyO was assessed by a cross to y1 w67c23 control in comparison to Su(H)Δ47 and Su(H)SF8 alleles. The expected 1:1 ratio of mutant and balancer chromosome CyO was observed, yet Su(H)LLL has a slightly lowered viability. (D) The new Su(H) alleles were crossed with Su(H)attP/CyO-GFP and the number of hemizygous pupae counted relative to the heterozygotes recognized by green fluorencence. Su(H)Δ47 and Su(H)SF8 served for comparison. (E) The homozygous control lines Su(H)gwt, Su(H)gwtΔi3, or Su(H)gwtΔi2i3 are like wild type, exemplified by a normal Wg staining. All three are able to complement the Su(H)attP allele: they are viable and have normal looking wing discs in hemizygosis. Likewise, Su(H)gwt-mCh appears wild type, whereas Su(H)LLL-mCh and Su(H)LLF-mCh display hypertrophied wing discs similar to the untagged strains (compare with Fig 2E’ and 2F’). Size bar represents 100μm in all panels. (F) Survival rates of the hemizygous controls Su(H)gwt, Su(H)gwtΔi3, or Su(H)gwtΔi2i3 over Su(H)attP was determined relative to wild type stock Oregon R (OreR). Inset numbers in (C), (D), (F) represents animals analysed. (TIF) [file pgen.1006774.s002.tif]

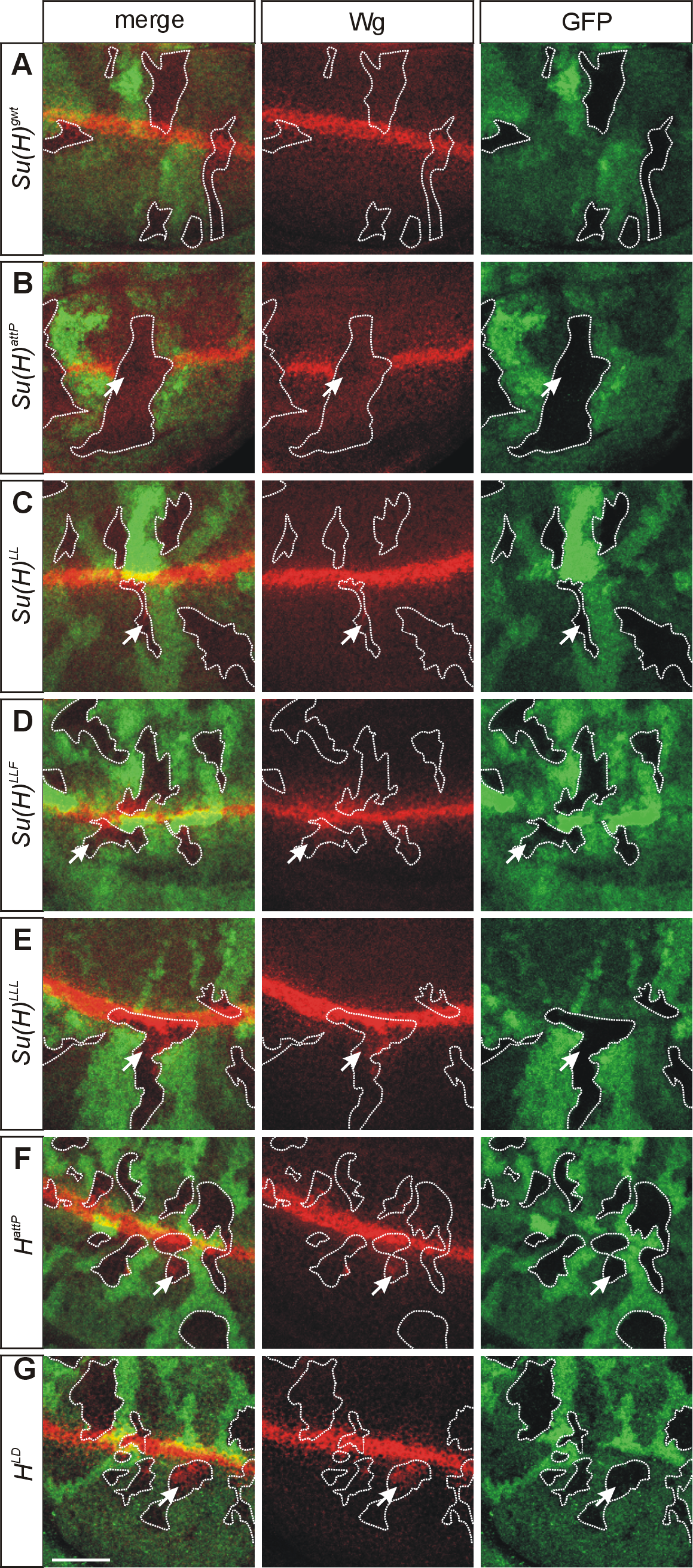

Supplement: S3 Fig — Expression of Wg (red) was analysed in cell clones homozygous mutant for the allele indicated. Mutant clones are within the central wing blade; they are marked by the absence of GFP (green) and are outlined. Examples of altered Wg expression are highlighted by arrows. (A) Wg is expressed along the dorso-ventral boundary of the disc. Control clones of Su(H)gwt do not disturb the wild type pattern. (B) Wg protein expression is lost from Su(H)attP homozygous cells (arrow) due to the absence of Su(H) protein. (C-E) A slight broadening of Wg expression is seen in cell clones homozygous mutant for any of the H-binding deficient Su(H) alleles, Su(H)LL (C), Su(H)LLF (D), or Su(H)LLL (E). (F-G) A similar broadening is observed in cells lacking H activity (arrow), as in HattP (F) or HLD (G). Size bar represents 25μm in all panels. (TIF) [file pgen.1006774.s003.TIF]

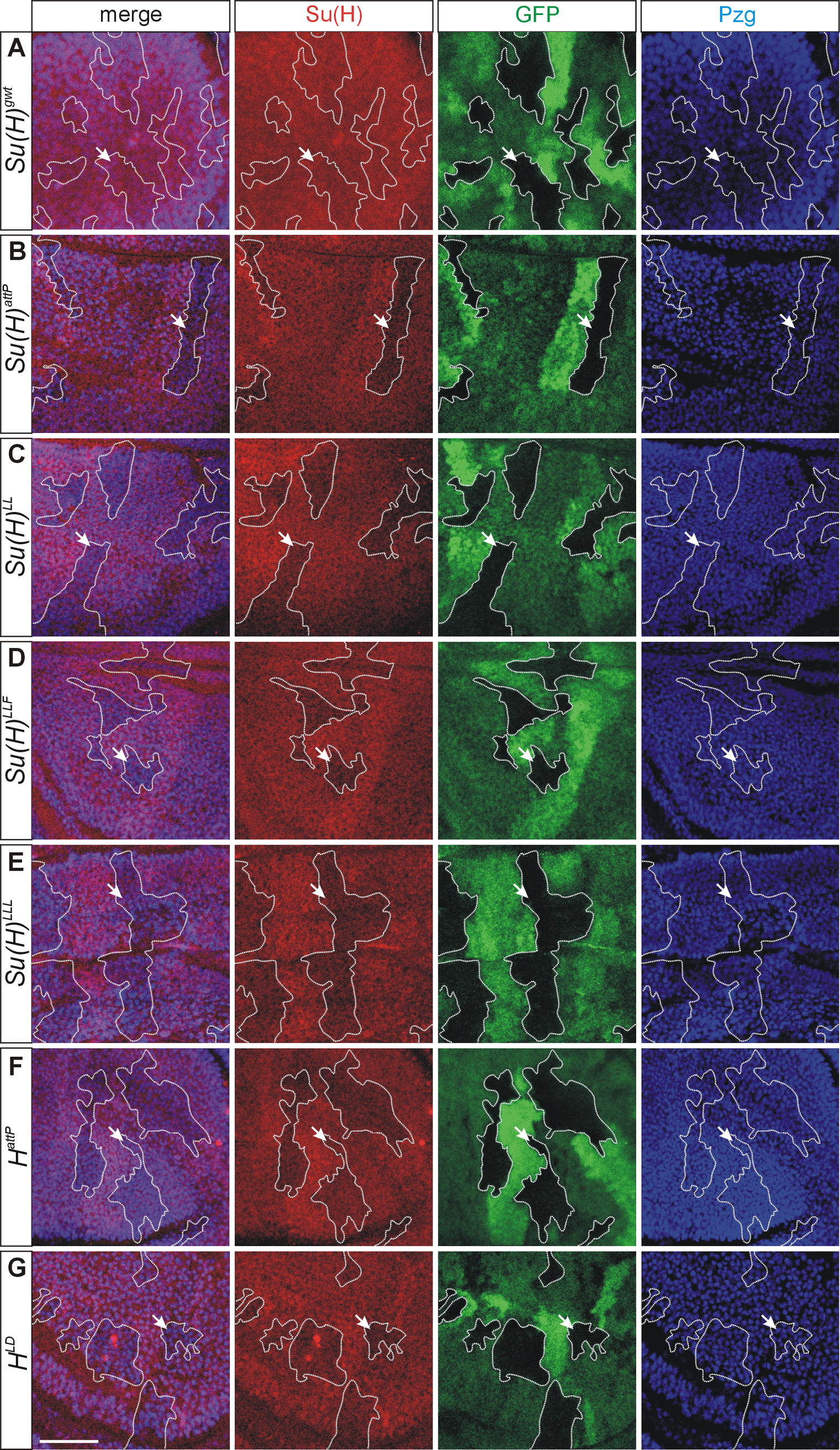

Supplement: S4 Fig — Clones were induced in wing imaginal discs; magnification of the central wing blade is shown. Genotypes are indicated on the left. Cells were stained with a polyclonal rat anti-Su(H) antiserum (red) [32], anti-GFP (green) as clonal, and anti-Pzg (blue) as nuclear marker [64]. Wild type homo- and heterozygous cells are marked by GFP; homozygous mutant cells lack GFP and are outlined for clarity (arrows point to examples). The merge shows an overlay of Su(H) and Pzg. (A) In contrast to Su(H)gwt control clones, (B) Su(H)attP null mutant cells show little Su(H) protein signals. (C-E) Note the reduction of Su(H) protein levels in H-binding deficient Su(H) mutant cells, (F,G) and alike in cells lacking H activity. (Bright dots in C,F,G are unspecific labelling). Size bar represents 50μm in all panels. (TIF) [file pgen.1006774.s004.tif]

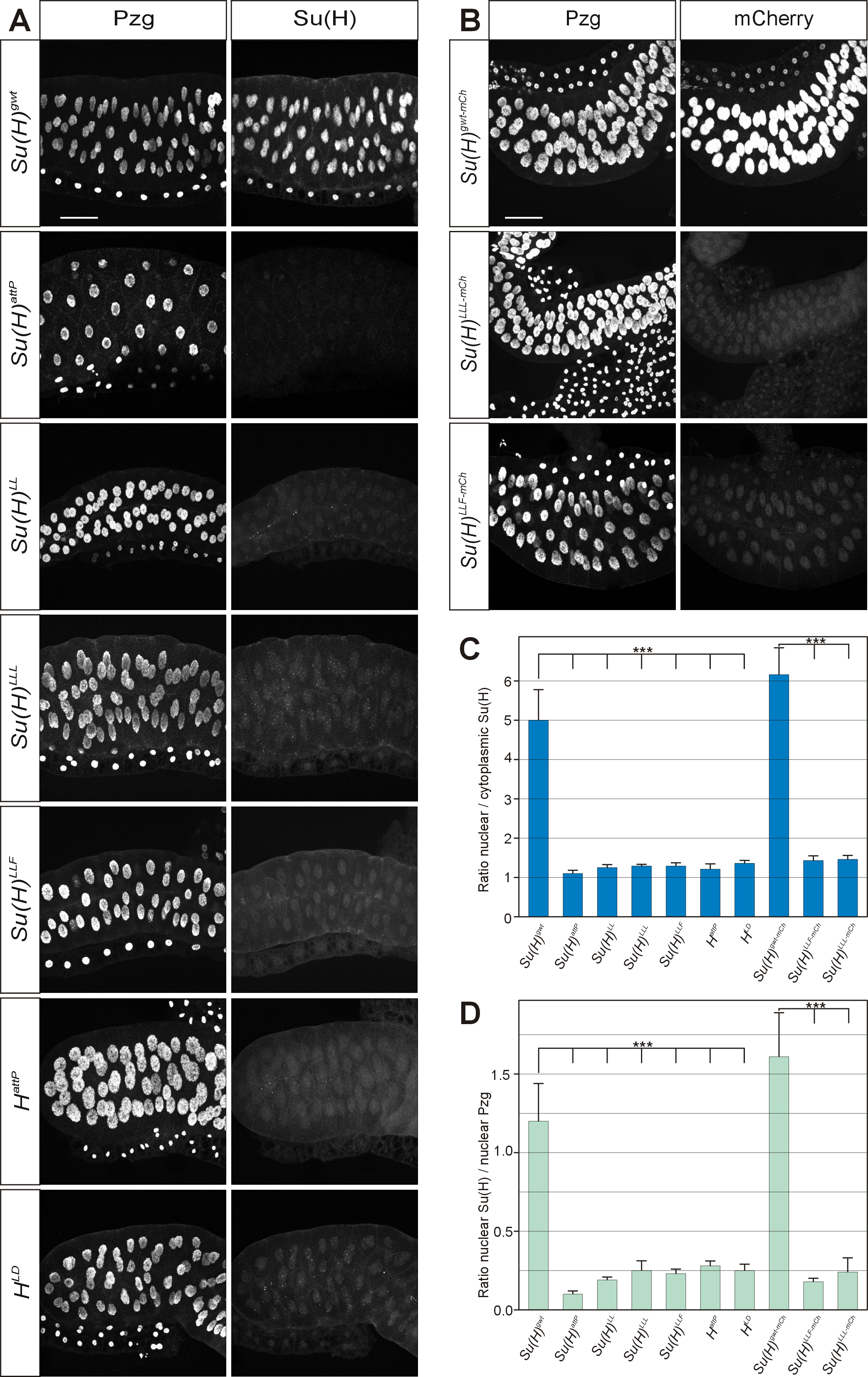

Supplement: S5 Fig — (A, B) Salivary glands from homozygous third instar larvae of the given genotype were doubly stained for Pzg as nuclear marker and for Su(H) and mCherry, respectively. Drastic reduction of nuclear Su(H) protein expression is apparent in the mutants. (C) Quantification of staining intensity was performed on stacks of pictures cutting through the entire gland using Image J. Intensity of all the nuclei from one gland was compared with the total cytoplasmic signal from that gland (n = 3–5). Loss of nuclear staining and no concurrent cytoplasmic enrichment is observed. (D) Signal intensity of Su(H) and Pzg staining of individual nuclei (25–50 per gland; 3–5 glands per genotype) were recorded using Image J. A four- to five-fold drop in Su(H) expression is seen in the H-binding deficient Su(H) as well as in the H alleles indicated. Differences between mutants and control are highly significant by ANOVA two-tailed Tukey-Kramer approach for multiple comparisons (***, p<0.001). Size bar represents 100μm in all panels. (TIF) [file pgen.1006774.s005.TIF]

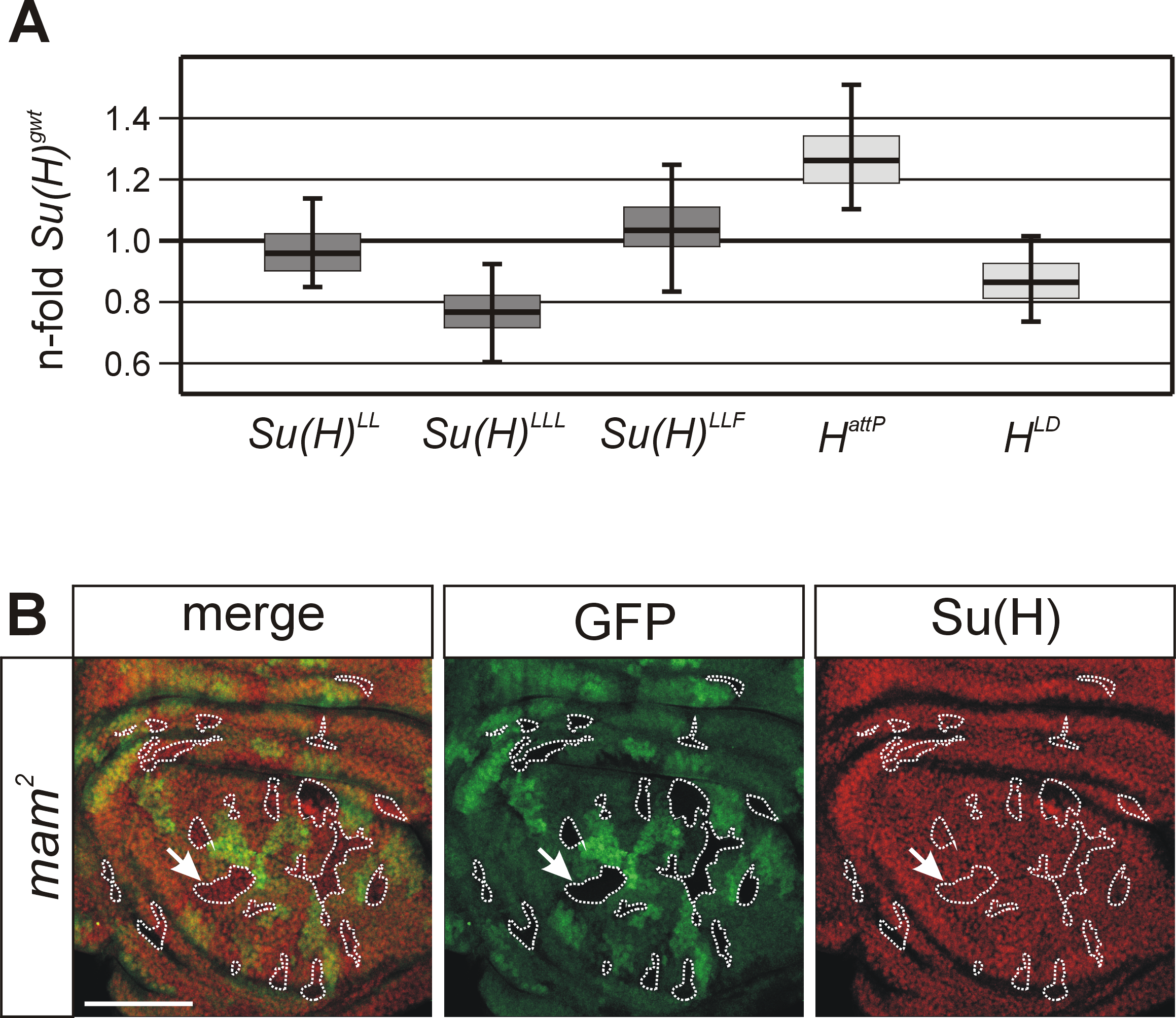

Supplement: S6 Fig — (A) Su(H) mRNA expression was quantified by qPCR relative to cyp33 and Tbp as reference genes. Efficiencies for Su(H) (0.97), for cyp33 (0.94) and for Tbp (0.92) were taken into account for the relative quantification [65]. mRNA was derived from homozygous mutant larvae and compared to Su(H)gwt. Data are assembled from three biological and two to four technical replicates. Mini-max depicts 95% confidence, median corresponds to expression ratio. Values are close to 1, i.e. close to Su(H)gwt expression; differences of about 20% between Su(H)gwt and HattP, or Su(H)gwt and Su(H)LLL are assessed statistically significant, however, might be attributed to stage variations. (B) Cell clones homozygous mutant for mam2, distinguished by the lack of GFP (green), show no changes in Su(H) protein expression (red). Arrow points to an example. Size bar represents 100μm. (TIF) [file pgen.1006774.s006.tif]

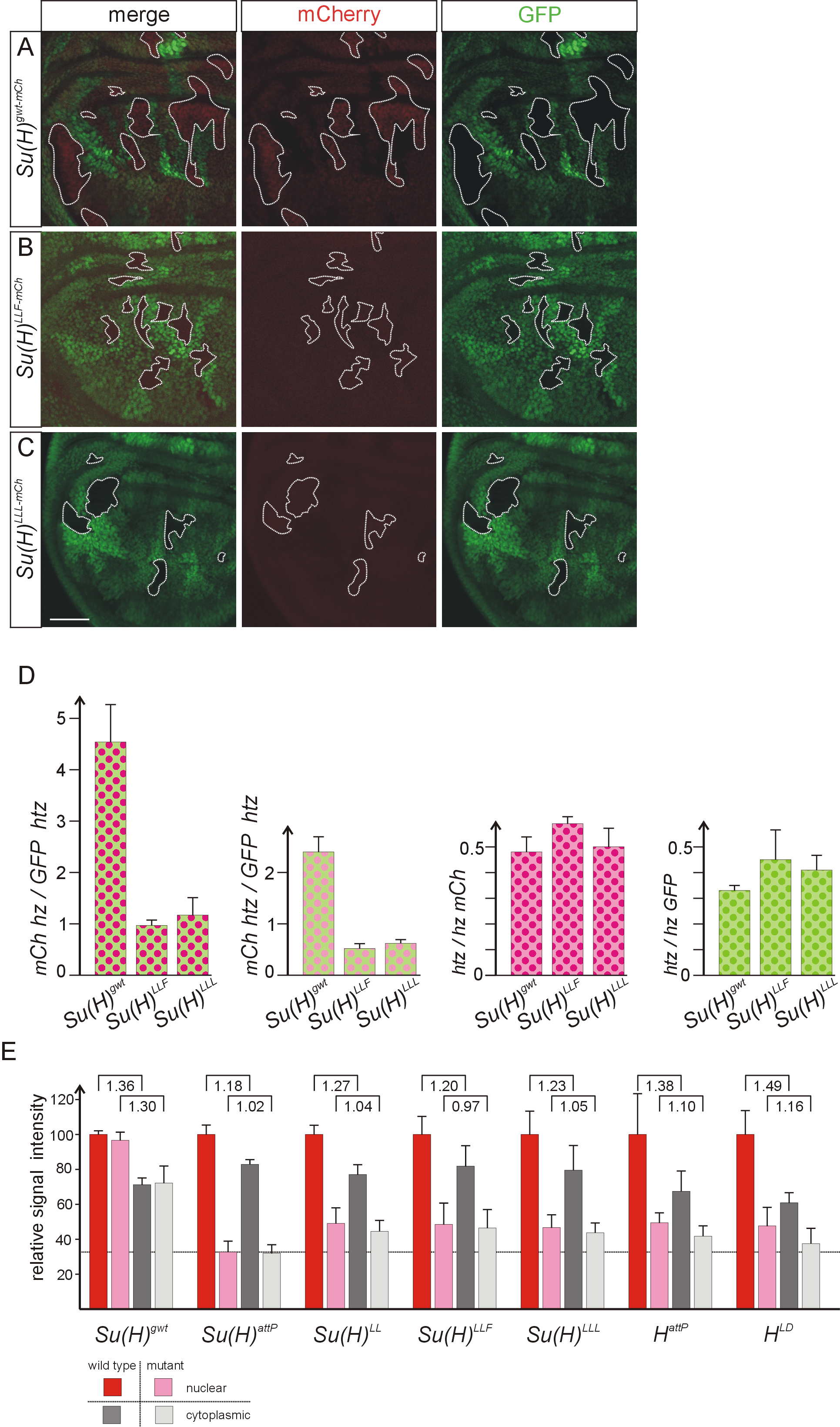

Supplement: S7 Fig — (A-C) Clonal analysis in Su(H)gwt-mCh, Su(H)LLF-mCh, and Su(H)LLL-mCh wing discs: red auto-fluorescence of mCherry is shown. Wild type cells are marked by GFP, displaying green auto-fluorescent GFP signals. Heterozygous cells show a weaker fluorescence signal of both. Clones are outlined for clarity. Size bar is 50μm. (A) The fluorescence signals of both the homozygous and heterozygous cells are clearly visible in Su(H)gwt-mCh clones. (B,C) In contrast, mCherry signals in either Su(H)LLF-mCh or Su(H)LLL-mCh homozygous cells are just above background, precluding signal quantification. Intensity of the red channel was increased to visualize the staining. (D) Signal quantification of Su(H)gwt-mCh, Su(H)LLF-mCh, and Su(H)LLL-mCh in clones stained with anti-mCherry and anti-GFP antibodies as in Fig 5A–5C. Signals were recorded at 20 points each within homozygous wildtype, mutant, and heterozygous clones at identical positions for mCherry and for GFP within each disc. To allow for comparison of intensity between control and mutant, homozygous (hz) or heterozygous (htz) mCherry signals were set in relation to GFP signals from heterozygous (htz) cells. Su(H)gwt-mCh protein is about four-fold enriched compared to Su(H)LLF-mCh or Su(H)LLL-mCh protein. Signal intensity of mCherry correlates well with gene dose (heterozygous is about half of homozygous, htz/hz), in contrast to the GFP signals, where the heterozygous signal is more than two-fold weaker than the homozygous signal. Error bars indicate standard deviation (n = 5–7 biological samples). (E) Quantification of nuclear versus cytoplasmic Su(H) protein detected with rabbit anti-Su(H) antibodies in clones as shown in Fig 4. Signals were recorded within and just next to 20 nuclei each within homozygous wild type (dark columns) or homozygous mutant clone (light columns) at identical positions within each disc. GFP signals were used to identify cell clones and Pzg signals to identify the nuclei. Grey values were determined wi [file pgen.1006774.s007.TIF]

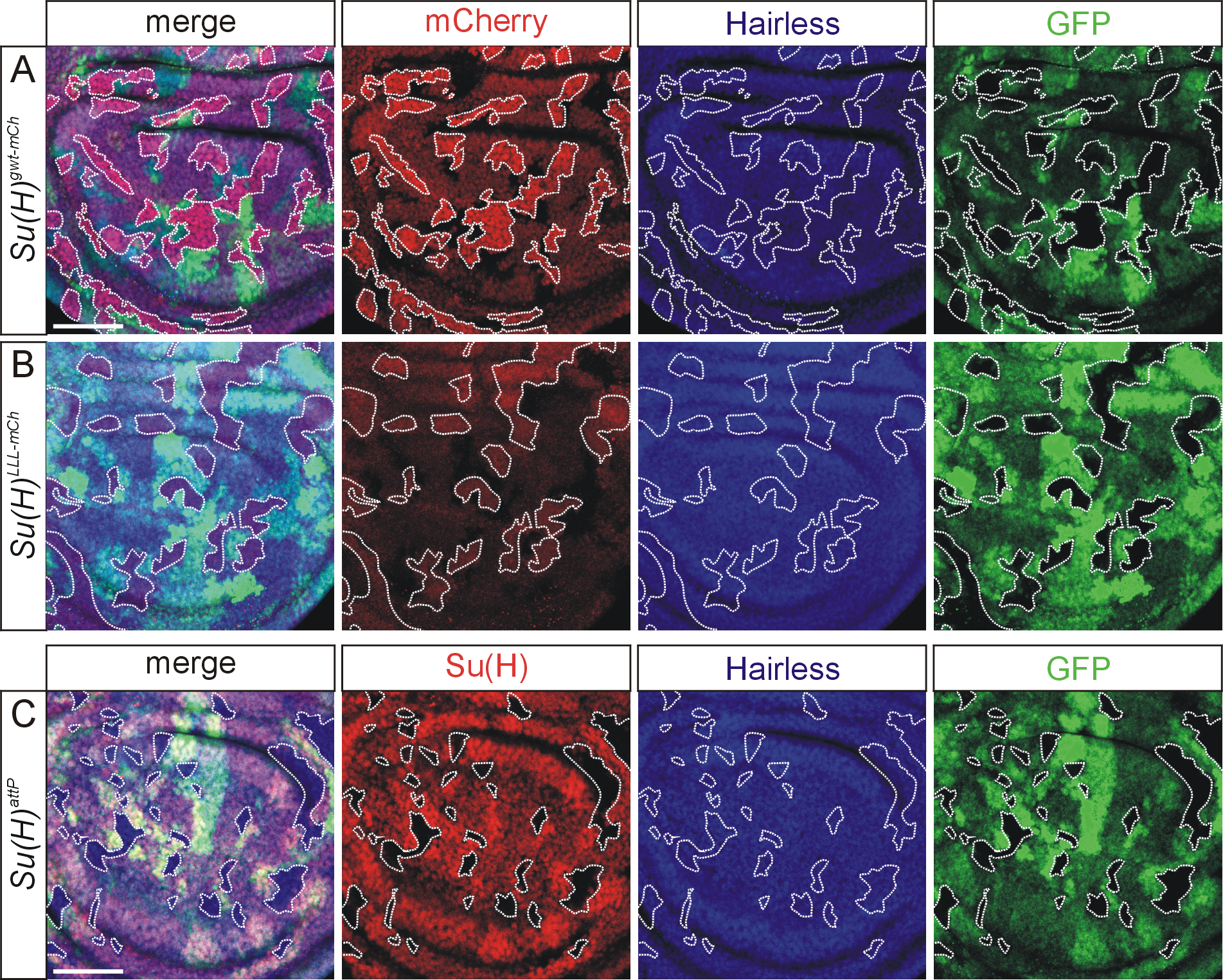

Supplement: S8 Fig — (A-B) Clones homozygous for Su(H)gwt-mCh (A) and Su(H)LLL-mCh (B) were induced in wing imaginal discs and stained for mCherry (red) and for H protein (blue). (C) Likewise Su(H)attP homozygous mutant cells lacking Su(H) protein were generated, and Su(H) protein (red) and H protein (blue) detected. Note equal distribution of H protein in all genotypes. Clones are marked with GFP [green in (A-C)]; homozygous wild type cells show high GFP levels, mutant cells lack GFP. The clones are outlined for clarity. Size bar represents 50μm in all panels. (TIF) [file pgen.1006774.s008.TIF]
